# Impacts of forest restoration on water yield: A systematic review

**DOI:** 10.1371/journal.pone.0183210

**Published:** 2017-08-17

**Authors:** Solange Filoso, Maíra Ometto Bezerra, Katherine C. B. Weiss, Margaret A. Palmer

**Affiliations:** 1 Chesapeake Biological Laboratory, University of Maryland Center for Environmental Science, Solomons, Maryland, United States of America; 2 National Socio-Environmental Synthesis Center, University of Maryland, Annapolis, Maryland, United States of America; 3 Department of Entomology, University of Maryland, College Park, Maryland, United States of America; University of Oregon, UNITED STATES

## Abstract

**Background:**

Enhancing water provision services is a common target in forest restoration projects worldwide due to growing concerns over freshwater scarcity. However, whether or not forest cover expansion or restoration can improve water provision services is still unclear and highly disputed.

**Purpose:**

The goal of this review is to provide a balanced and impartial assessment of the impacts of forest restoration and forest cover expansion on water yields as informed by the scientific literature. Potential sources of bias on the results of papers published are also examined.

**Data sources:**

English, Spanish and Portuguese peer-review articles in Agricola, CAB Abstracts, ISI Web of Science, JSTOR, Google Scholar, and SciELO. Databases were searched through 2015.

**Search terms:**

Intervention terms included forest restoration, regeneration/regrowth, forest second-growth, forestation/afforestation, and forestry. Target terms included water yield/quantity, streamflow, discharge, channel runoff, and annual flow.

**Study selection and eligibility criteria:**

Articles were pre-selected based on key words in the title, abstract or text. Eligible articles addressed relevant interventions and targets and included quantitative information.

**Results:**

Most studies reported decreases in water yields following the intervention, while other hydrological benefits have been observed. However, relatively few studies focused specifically on forest restoration, especially with native species, and/or on projects done at large spatial or temporal scales. Information is especially limited for the humid tropics and subtropics.

**Conclusions and implications of key findings:**

While most studies reported a decrease in water yields, meta-analyses from a sub-set of studies suggest the potential influence of temporal and/or spatial scales on the outcomes of forest cover expansion or restoration projects. Given the many other benefits of forest restoration, improving our understanding of when and why forest restoration can lead to recovery of water yields is crucial to help improve positive outcomes and prevent unintended consequences. Our study identifies the critical types of studies and associated measurements needed.

## Introduction

Water security is tightly linked to the availability of both surface water and groundwater stores directly accessed for human consumption (blue water) and water that resides in the unsaturated soil layer and vegetation canopy (green water) [1]. Plant production depends heavily on green water and is responsible for about 60% of the global food supply [2]. However, because blue water also contributes substantially to agricultural production and is critical for the support of wildlife and the provision of drinking water, there is great concern that growing demand has resulted in up to 2.9 billion people facing shortages of blue water for 3 to 6 months of a year [3]. For this reason, conservation efforts are focusing extensively on how to recover or retain existing water resources, including delivery to surface waters.

Unfortunately, land use activities have transformed most of the planet’s land surface [4], and a great deal of land conversion has come at the expense of forests [5]. This has great implications for water resources [6], such as the loss and degradation of surface waters that humans rely upon heavily [7–11]. Perhaps because of this well-known pattern and the fact that forest protection efforts are associated with water provision and purification services [12], a common perception among managers, policy makers, and the public is that reforesting or forest expansion will help mitigate water problems [13]. Not coincidentally, a growing number of forest restoration projects worldwide have integrated water resources management and policies [12,14].

While it is reasonable to expect a positive effect of forest restoration on water quality since comparisons of water quality across land uses have repeatedly shown that forest lands provide the highest quality of surface waters [15], the impacts of forest cover expansion on blue water quantity are at best unclear [13,16,17]. In fact, many assessments of the impacts of forest cover expansion on the water balance of watersheds have reported reductions in annual runoff [18–20], especially in drier regions and in areas where forests have replaced grasslands or shrublands [21]. Despite these reports, the debate continues over whether or not forest restoration can help recover water provision functions and improve surface water yields in watersheds [22].

Evapotranspiration (ET) is a major component of the water balance in terrestrial ecosystems and is well known to influence regional water availability [23]. Forests have relatively high ET rates in comparison to most other land use and cover types [24], which is why water yields usually decrease upon the conversion of different land uses into forests. However, because ET rates decrease with forest age [25], time since the implementation of forest restoration projects may play an important role in determining if, and when, forest restoration will increase water yields.

Many have recently raised the issue of restoration scale due to the pivotal role that forests play in supplying atmospheric moisture that becomes precipitation [17], especially in the tropics [26]. When implemented at a large spatial scale, some researchers have argued that forest restoration can enhance water yields by increasing rainfall via atmospheric feedbacks [17]. However, there is no empirical evidence supporting this notion. Given their goals and motivations, forest restorations are commonly implemented at small scales [27], which limits opportunities to test this proposition. Further, enhanced precipitation from large-scale restoration may simply intensify the water cycle [28], increasing the availability of water for plants, but not necessarily resulting in higher water yields for people or other services. However, if the physiological responses of plants to increasing levels of carbon dioxide and temperatures result in reduced transpiration rates and higher water-use efficiency [29], large-scale restoration in certain regions of the world may lead to higher water yields [30,31]. Clearly, a multitude of possible interacting factors can influence hydrological response of forest cover restoration, and most of these are rarely considered in studies of forest restoration outcomes. These include for example land-use history, ecological conditions, tree species, forest type and management, and the type of method used to increase forest cover [19,32,33]; even the changing climate may play a role [34].

Given the growing number of projects integrating forest restoration and water management, the primary objective of this systematic review was to provide an assessment of the state-of-the-science regarding the effects of forest cover expansion and restoration on water yields. Additionally, this study aimed to provide a quantitative analysis of the information available to determine if hydrologic outcomes from forest restoration differ from other types of forest cover expansion. Our secondary objective was to determine if the scientific information available in the literature is adequate to generalize results across different settings and regions of the world. To do this, we tracked (1) the type of forest cover change or intervention, (2) the method of water quantity data acquisition, (3) the spatial scale of the study watershed, (4) the temporal scale or age of the study, (5) the geographic location of the study, and (6) the dominant climate of the study region for each study used in our assessment. This allowed us to determine if, where or how forest projects and studies were done could be influencing observed water outcomes.

## Methods

### Systematic literature review guidelines

We defined a clear goal and a set of questions(s) for the review using a formal methodology for conducting systematic reviews in the conservation and environmental sciences [35]. The over-arching goal was to evaluate the extent of scientific consensus concerning the impacts of forest restoration on water quantity.

The literature search was conducted using key words or search terms. Accessible sources included academic databases and search engines that provided comprehensive coverage of peer-reviewed publications. The sources included Agricola, CAB Abstracts, ISI Web of Science, JSTOR, and Google Scholar. The search engine SciELO was also used to increase access to Portuguese publications from Brazil where there has been an extensive focus on forest restoration. Databases were searched from 1953 through 2015 and regardless of citation rate.

The search process, adapted from [35], was based on the use of key words or search terms either in the title, the abstract or both. The key words and search terms were identified after an initial exercise exploring intervention and target terms that would yield the largest number of articles. We began with the terms “forest restoration” and “water yield,” however, the search was broadened significantly as these terms did not result in many articles. Complementary target terms such as “streamflow,” “stream flow,” “discharge,” “annual flow,” and “channel runoff” were added as indicators or synonyms for water yield/quantity. Complementary target terms for “forest restoration” included “afforestation/forestation,” “reforestation,” “forestry,” “forest regrowth,” “forest regeneration,” and “forest secondary growth” (Table 1). Use of the terms “afforestation/forestation” and “reforestation” were based on the assumption that they can represent a form of active forest restoration; “forestry” was assumed to represent the practice of managing for forests; “forest regrowth,” “forest regeneration,” and “forest secondary growth” were added for representing a passive form of forest restoration.

**Table 1:** Categories and search terms or key words used in the literature review search process.

Categories	Terms or Key Words
Subject	Watershed, catchment, river, stream, surface water.
Intervention type	Forest restoration, reforestation, afforestation, forestation, forestry, forest regrowth, forest regeneration, forest secondary growth.
Target	Water yield, water quantity, streamflow, stream flow, discharge, annual flow, channel runoff.
Complementary target terms	Baseflow, peak flow, flood, flooding, groundwater, soil infiltration, infiltration capacity.

### Article selection criteria

Articles were first pre-selected (by one reviewer) based on whether they contained key words in the title, abstract, or body of the text, and retained if they addressed the central review question (by two reviewers).

Articles selected for use in the review had to have at least one case study or information that fulfilled each of the following criteria: 1) the study addressed a process of forest cover expansion or restoration including one of the intervention types (Table 1); 2) the study presented data on one or more of the target indicators (Table 1) for at least one well-defined watershed or catchment; and 3) the study documented changes in water yield or quantity that occurred (a) during a period of time following processes of forest cover restoration or expansion, (b) in watersheds with different land cover and/or stages of forest growth (i.e., used space for time study design), or (c) in watersheds with contrasting forest cover, including deforested and fully reforested watersheds (paired catchment design).

Articles were excluded if they only assessed the effects of deforestation, reported only on changes in surface runoff (i.e., overland flow) to forest cover expansion, provided no data on channel runoff, or involved irrigation. Articles that only provided a qualitative assessment were also excluded.

### Literature and data organization

To accomplish our primary objective of synthesizing the quantitative data in the literature relevant to hydrologic response to forest expansion, the pre-selected papers were stored in the reference-managing software Mendeley (v1.16.3) and assigned qualitative attributes through a “tag” function. Each paper was attributed multiple tags representing different qualitative attributes, which were systematically assigned according to the parameters in Table 2. Additional parameters and tags were used to select a subset of papers or study cases that could be used in a meta-analysis to address our secondary objective to evaluate if attributes of the studies may be influencing the water outcomes reported across the literature.

**Table 2:** Parameters and tags used in the systematic classification of papers or case studies selected (in order of relevance). Parameters 11 and 12 were used to select a subset of case studies to address the secondary objective.

Parameter	Tags/ Qualitative Attributes
1. Type of response of forest restoration or other reforestation processes on water yield	Positive/negative/neutral/mixed
2. Intervention type	Forest restoration; reforestation; afforestation; forestation; forest regrowth/regeneration; forestry; mixed; undefined
3. Intervention type group	Forest restoration explicit; forest restoration implicit; forestry; mixed
4. Type of forest	Native forest; non-native forest; mixed forest; undefined
5. Method of data acquisition	Empirical [data]; model [data]; historical [data]
6. Spatial scale of study	Catchment area (km^2^): (≤ 1); (> 1 and ≤10); (> 10 and ≤ 100); (> 100 and ≤ 1000); (> 1000 and ≤ 10000); and (> 10,000)
7. Duration of data collection	Number of years of study: 1 to >150 y
8. Geographic region	Africa; Middle East; Asia; Oceania; Europe; North America; Central America; South America
9. Dominant climate of the study region	Köppen-Geiger climate classes derived from study sites coordinates
10. Other types of hydrological data that assess effects of forest restoration or reforestation processes besides surface water yield	Flood frequency/peak flows decrease/increase; baseflow increase/decrease; groundwater level increase/decrease; infiltration capacity increase/decrease; soil infiltration increase/decrease
11. Type of water yield data	Surface water yield/other; channel runoff
12. Study design	BACI (Before-After-Control-Impact) experiments

After the final selection and completion of the systematic classification in Mendeley, the meta-data associated with the papers was transferred to an Excel spreadsheet, where each column represented a tag name (Table 2). The responses to the tags were represented as a one (1) indicating that a particular result was reported for the paper, or, a zero (0) indicating the opposite result or that there was no result accounted for in the study.

When a paper provided water yield information for multiple catchments or watersheds (except for synthesis papers), information from each catchment or study case was individually extracted and tagged accordingly (S1 Table). Only information from the intervention catchment, not the control, was extracted from studies that used paired catchments or a space-for-time approach. Data from the study cases that met the criteria for addressing the secondary objective were tagged according to parameters 11 and 12 (Table 2) and analyzed separately.

Geographic coordinates for each study site were added based on the information provided in the papers. If a study site’s latitude and longitude were not provided, we obtained the coordinates using Google Earth based on the location of the study site described in the paper.

As climate information was not standardized across papers, we classified the study cases for climate based on the geographic position of the study site using a GIS raster of the global Köppen-Geiger climate map (version June 2006 obtained at http://koeppen-geiger.vu-wien.ac.at/present.htm). Climate information was extracted for each study case using ArcGIS Desktop version 10 (ESRI, Redlands, CA: Environmental Systems Research Institute).

The qualitative attributes used to determine how water yield and/or its derivatives responded to forest cover expansion were defined as positive (yield increased), negative (yield decreased), neutral, or mixed (yield did not change compared to its control site or there were multiple responses, generally because water yield first increased and later decreased with forest cover change).

### Rationale for parameters and tags used in classification

The qualitative attributes used for the classification were based on the description and terms provided in the articles selected in the review. For type of intervention, exact term(s) provided in the articles were used (e.g. reforestation, afforestation/forestation, forest regrowth, forestry, forest restoration), but, because terminology was largely inconsistent and sometimes included more than one term, additional tags were used based on whether or not the intervention had explicit or implicit restoration goals. These tags were used to group the selected studies into major groups of intervention (“forest restoration—explicit or implicit”, “forestry,” and “mixed”). Studies in the “explicit forest restoration” group explicitly called the intervention forest restoration or clearly stated the intention of reinstating ecological processes or recovering forest structure, ecological functioning, or biodiversity. Studies in the “implicit forest restoration” involved abandonment of agricultural land with subsequent forest regeneration or suggested restoration goals without calling the intervention forest restoration. Studies in the “forestry” group stated or suggested goals such as timber production, silviculture, or agroforestry. The “mixed” group included studies combining forest restoration with forestry. The rationale for the use of these groups is that the type of intervention is likely to affect the capacity of forests to recover hydrological processes that sustain water yields. Since forestry is more likely to involve intensive management and frequent cutting, studies involving forestry may be more prone to result in negative water yields compared to studies involving interventions such as regeneration, second growth, regrowth, or other types that we included in the forest restoration category.

The parameter “type of forest” was used to examine whether or not the water yield response to forest cover expansion in a watershed was associated with the presence or absence of native trees. Several studies have shown that water demand can vary substantially among tree species [36–38] and that native species may be more adapted to water stress than non-native trees [39]. Hence, the volume of water available for groundwater recharge and surface water yield may be higher in watersheds restored with native trees and may result in fewer negative water yield responses following forest cover expansion.

The method of data acquisition (i.e., empirical data or model) was also classified for each study, since this may have an influence on the water yield outcomes of the study. Hydrologic simulation models are inexact representations that mimic the movement of water in the physical environment [40], especially if the model is not calibrated.

Since spatial scale of the study is thought to influence the water yield outcome [17,41], we classified each selected paper or case study into six classes spanning from < 1 km^2^ to > 10,000 km^2^ to determine the influence of spatial scale on the frequency of positive versus negative water yield results. Studies reporting results from plot-scale measurements were excluded from the selection. Likewise, we examined the influence of temporal scale on the outcome of the studies selected by categorizing them according to the number of years that water yield data were collected or produced, from one to > 100 years.

Studies were classified according to the duration of the water yield data collection period in order to ask if study duration could be a factor influencing the distribution of negative versus positive water yield outcomes after the onset of forest growth or regrowth. Forest age would have been a more appropriate parameter to use, but information about forest age was rarely provided. The meta-analysis using a subset of studies provides additional information about the potential influence of time scale on water yields, which should compensate for the limitations in the general analyses.

Numerous studies assessed the impacts of forest cover expansion on water using other hydrological data in addition to direct water yield measurements. Such studies were received additional attributes with specific responses (Table 2), which were analyzed separately.

The secondary objective was addressed by analyzing only a subset of papers based on type of water yield data reported and study design, as represented by parameters 9 and 10 (Table 1). These parameters were used to facilitate data comparison and a meta-analysis. Geographic representativeness was also important, so a pre-requisite for these papers was that they covered a wide range of geographic locations and climate.

### Assessment of the systematic classification

Before data from the studies could be synthesized and analyzed, the systematic classification was cross-checked for consistency of identification and categorization using an intercoder agreement process, a statistical method recommended for systematic reviews in environmental management [42] to demonstrate consistency among observational ratings from multiple reviewers. The intercoder agreement method consists of re-classifying and re-tagging about 20% of the selected papers by a reviewer who did not participate in the initial systematic tagging and classification process to test for agreement with the classification of the first reviewers. In our case, the agreement was verified for the ‘type of water yield response to forest cover expansion’ (i.e., positive/negative/neutral/unclear) in the papers and study cases selected by the first two reviewers, as described above. When the initial coding yielded poor agreement among reviewers, as is often the case [43], adjustments were made and the procedure repeated until the agreement level reached at least 80%, denoting the likelihood that other researchers can independently replicate the results of the review. Bias in the level of agreement was tested by using the Cohen's kappa coefficient [44].

### Data synthesis and analysis

For the first objective, data from the studies were synthesized by using counts and proportions, and the distribution of water yield responses from studies according to the type of forest cover expansion. To investigate for potential bias generated from the availability of studies in the literature, we also determined the percentage of cases within each category of attributes for the parameters listed in Table 2 (2 to 8). We used functions of the *dplyr* package in R to determine the frequency of cases according to the analysis of interest. A complete summary of the articles and data in the systematic review is available in the supporting information section (S1 Table).

The meta-data extracted from the subset of papers selected to address the secondary objective were used to further explore associations between the water yield responses reported and temporal or spatial scales of the studies. The strength of the associations was evaluated using Pearson's correlation.

To assess the degree to which the selected studies represent an unbiased sample in terms of geographic position, we conducted a geographic representativeness analysis. First, the geographic coordinates (latitude and longitude) for each case study was entered manually on a Google Earth world map to create a visual analysis of the distribution of the studies available in the literature. Subsequently, the total number of studies were computed per major continental region (Africa, Asia plus Middle East, Oceania, Europe, North America, and Central America plus South America; Table 2) and then normalized by dividing the number of studies by the total area of the region.

## Results

### Studies identified and quantitative analysis

**Fig 1:**
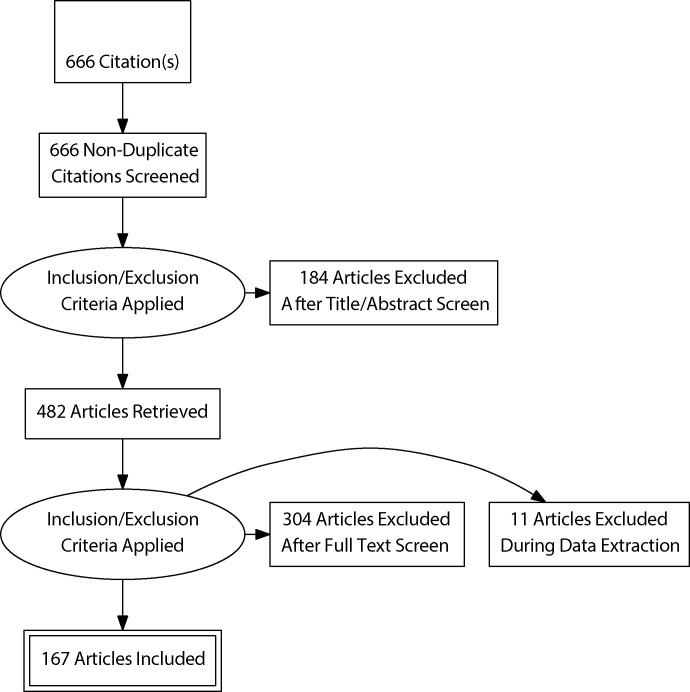
PRISMA flowchart.

The initial search for scientific literature on the effects of forest cover expansion on water yield identified a total of 666 papers, including peer-reviewed journal articles, conference proceedings, and book chapters (Fig 1). Out of this, 482 were deemed relevant to the main systematic review question based on their abstracts. Of these, 199 were selected for having data on water yield or derivatives following forest restoration or other forms of forest cover expansion, and 167 were retained during data extraction. Direct metrics of water yield included annual flow or stream/river channel runoff and baseflow or low-flow. Indirect metrics or water yield derivatives included flood frequency or magnitude, groundwater level or recharge, and water infiltration or percolation into soils.

**Fig 2:**
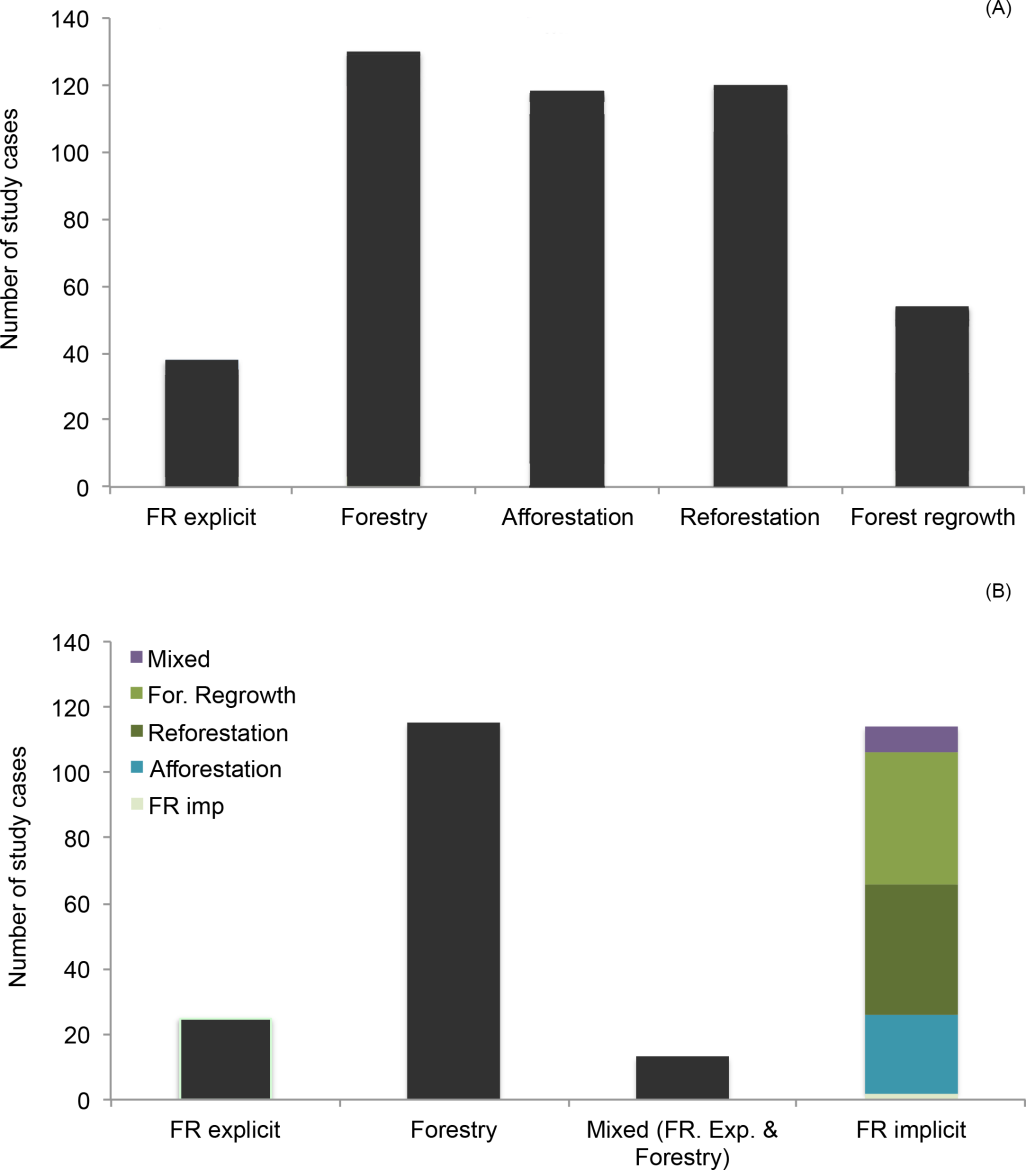
Studies by type of intervention. Percent distribution of study cases according to type of intervention described in the papers (a) and type of intervention based on the reclassification (b). Only study cases reporting on a direct measures of water yield are included (*n* = 308).

Most of the papers selected contained more than one case study. Therefore, the 167 papers included a total of 308 individual study cases that could be used to evaluate the impacts of forest cover expansion on water yields (S1 Table). A small number of studies were explicitly based on restored forests; most were based on forestry systems (Fig 2A). However, many studies based on reforestation, forest regrowth, and afforestation qualified as implicit restoration in the reclassification process described above, increasing the number of forest restoration cases (Fig 2B).

**Fig 3:**
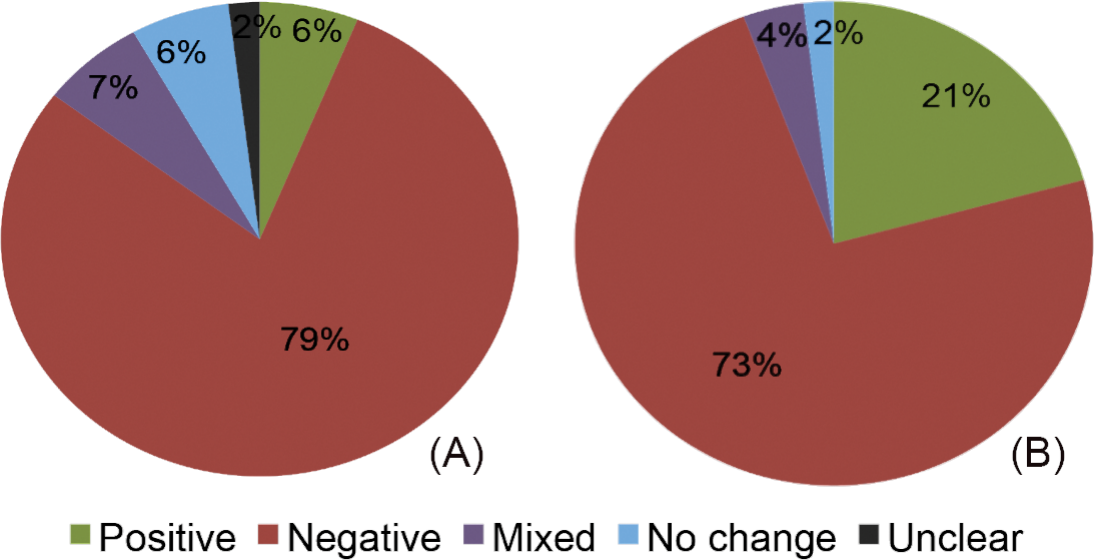
Water yield responses measured directly. Percent distribution of results in study cases that directly measured changes in annual water yield/channel runoff (*n* = 308) (a), and baseflow (*n* = 53) (b).

In about 80% of all study cases reporting on annual water yields, the effect of forest restoration and other forms of forest cover expansion was negative (i.e., yield decreased), while in 6% the effect was positive (Fig 3A). The remaining studies reported no change (7%), mixed (5%), or unclear effects (2%). Changes reported for baseflow were similar, with the majority of cases showing a decrease (63%) (Fig 3B). However, the number of cases with baseflow data was small by comparison.

**Fig 4:**
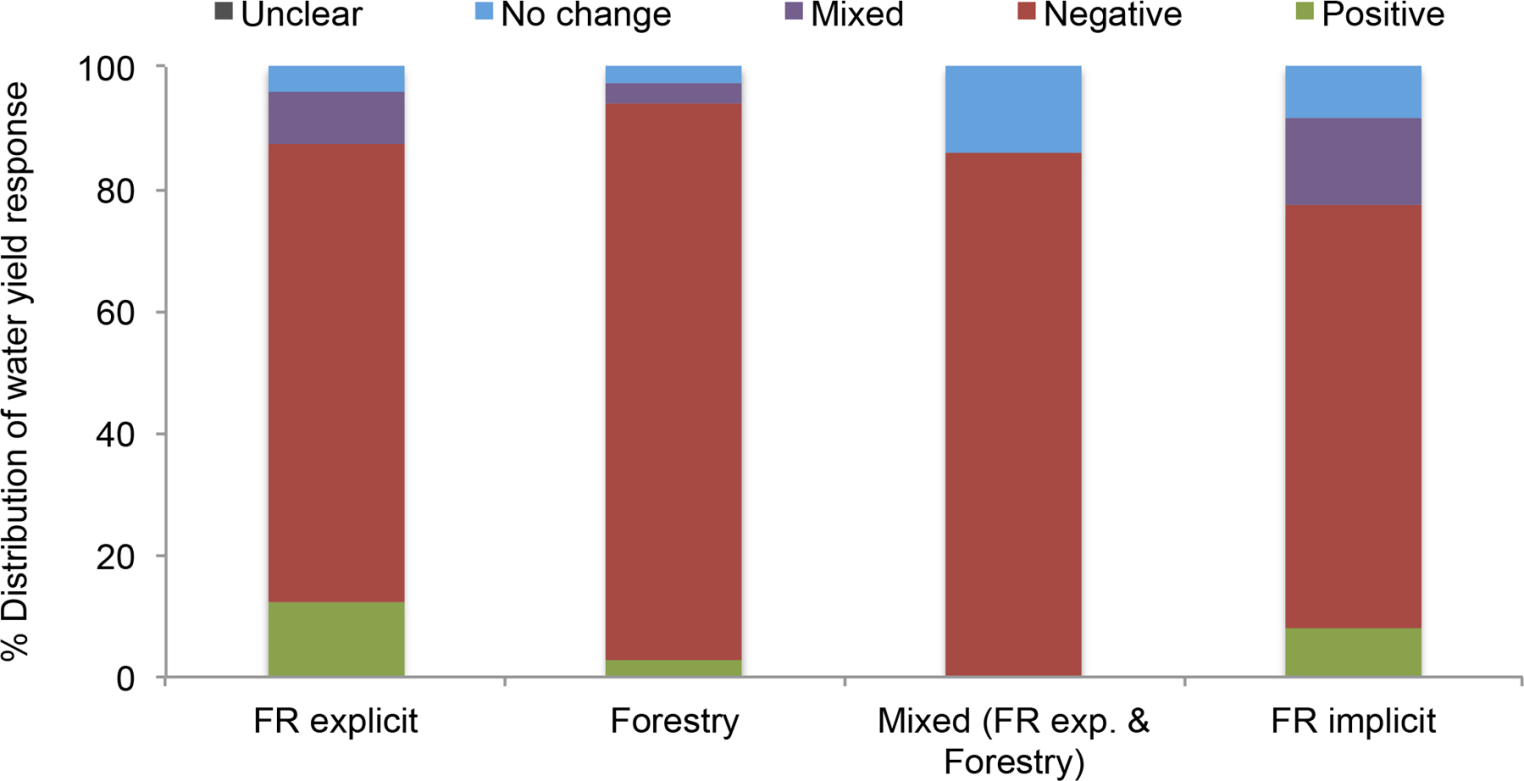
Water yield by type of intervention. Percent distribution of direct metrics of water yield responses according to intervention type group.

Water yield reduction following forest cover expansion was the dominant response in the study cases regardless of the type of intervention in the reclassification scheme (i.e., forest restoration explicit and implicit, forestry, and mixed) (Fig 4). However, the percentage of positive results in cases that qualified as implicit forest restoration was slightly higher.

**Fig 5:**
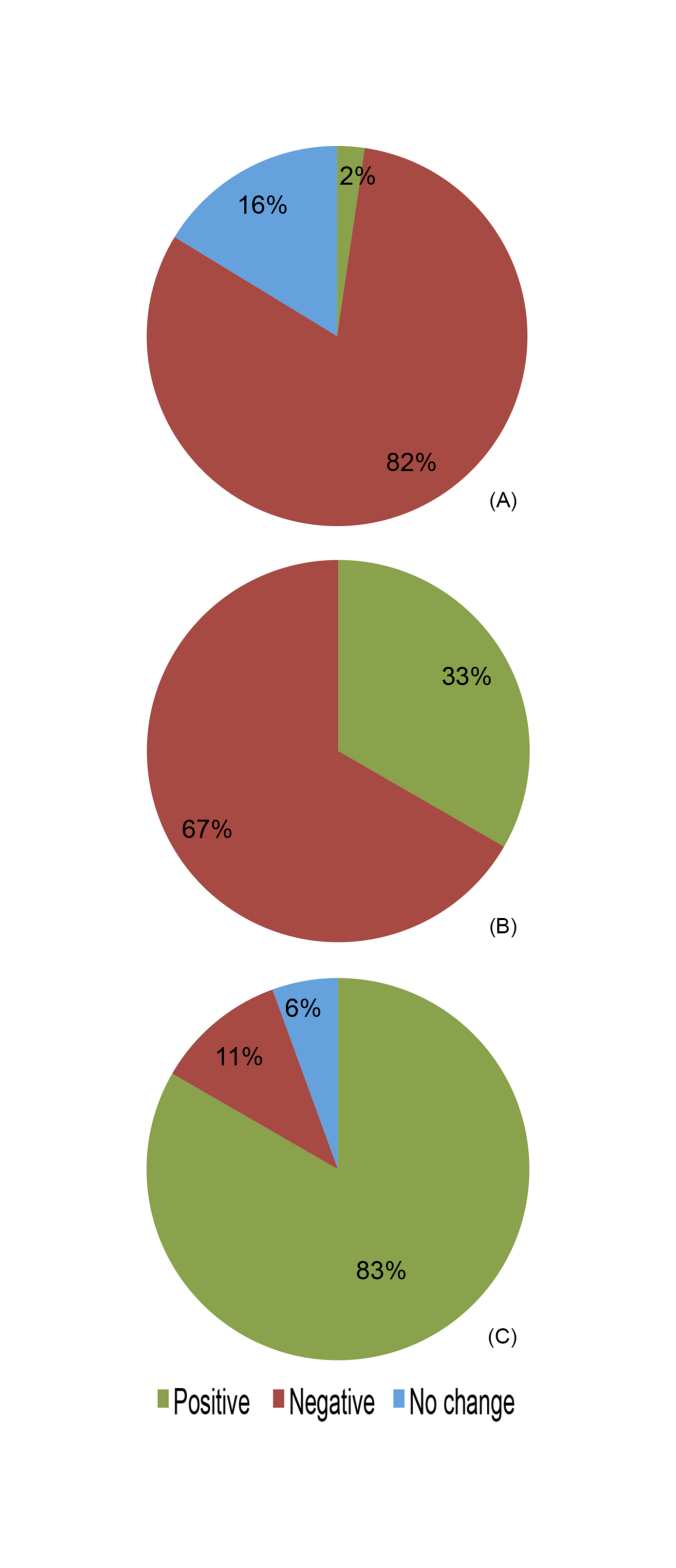
Water yield responses measured indirectly. Percent distribution of study cases with a positive, negative, or neutral effect of forest cover restoration or expansion on indirect water yield metrics, including (a) flooding frequency or magnitude of peak flows (*n* = 43), (b) groundwater level or recharge (*n* = 15), and (c) infiltration capacity or soil infiltration (*n* = 18).

A relatively small number of studies reported on indirect metrics of water yield (*n* = 43). In 82% of the studies with data on peak flows or flooding frequency (Fig 5A) and in 67% of the ones with data on groundwater levels or recharge (Fig 5B), the results were negative (i.e., flooding or peak flows and groundwater decreased with forest cover expansion). In contrast, in 83% of the studies reporting on infiltration capacity or soil infiltration the result was positive, (i.e., infiltration increased) (Fig 5C).

### Potential explanatory factors

**Fig 6:**
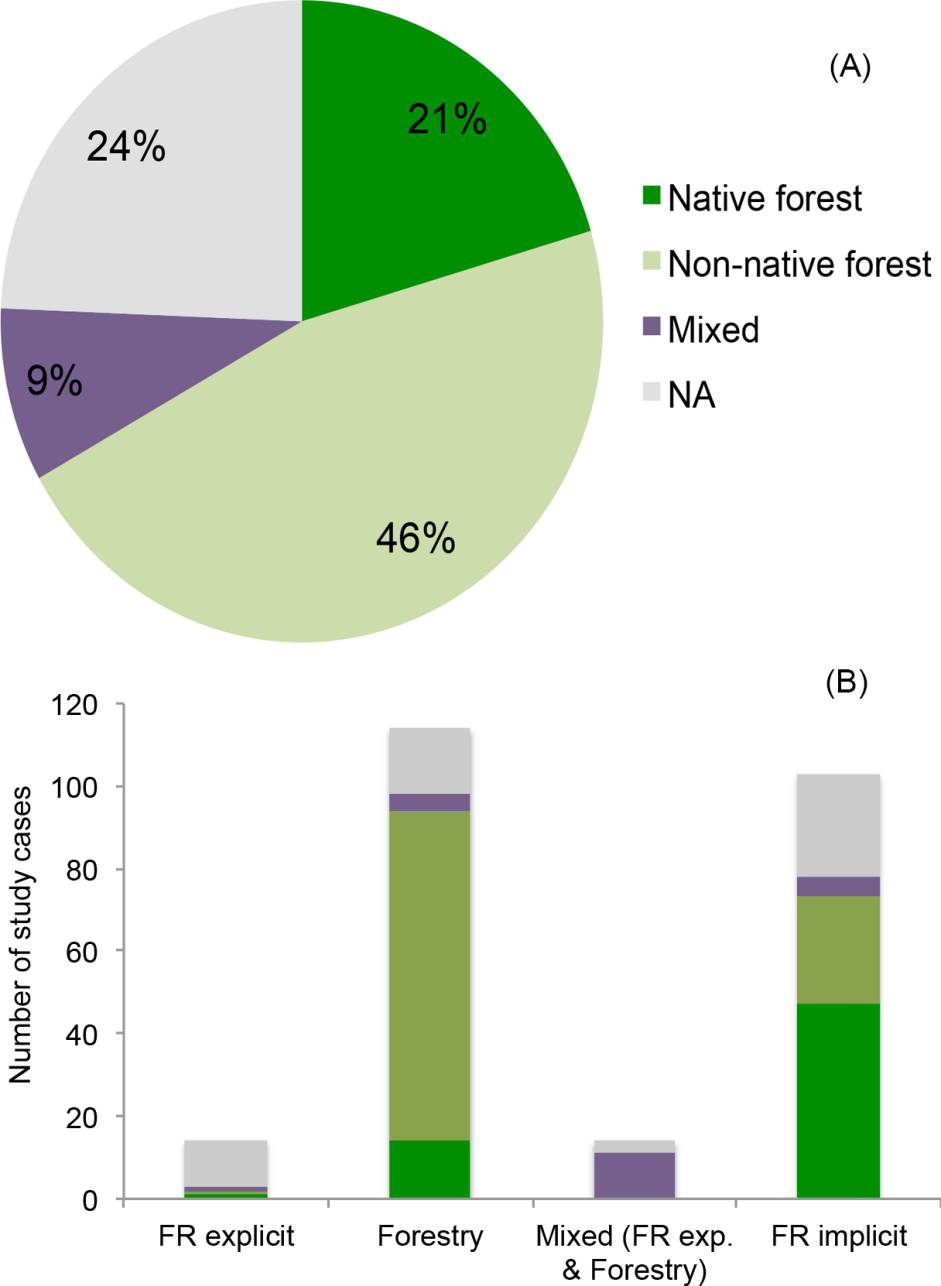
Distribution of studies by forest type. Percent distribution of native, non-native, and mixed forest types among all studies selected in the review (a) and within each group of forest intervention (b). The results are based on study cases reporting on a direct measures of water yield (*n* = 308).

As indicated above, the majority of studies available in the literature and selected in the review were based on “forestry” and not on “explicit forest restoration” projects. The studies were also predominantly based on forests stands with non-native tree species as opposed to native species (Fig 6A). Studies classified as implicit forest restoration had a relatively larger percentage of cases with native species (Fig 6B). Most studies classified as explicit forest restoration did not report on forest type.

**Fig 7:**
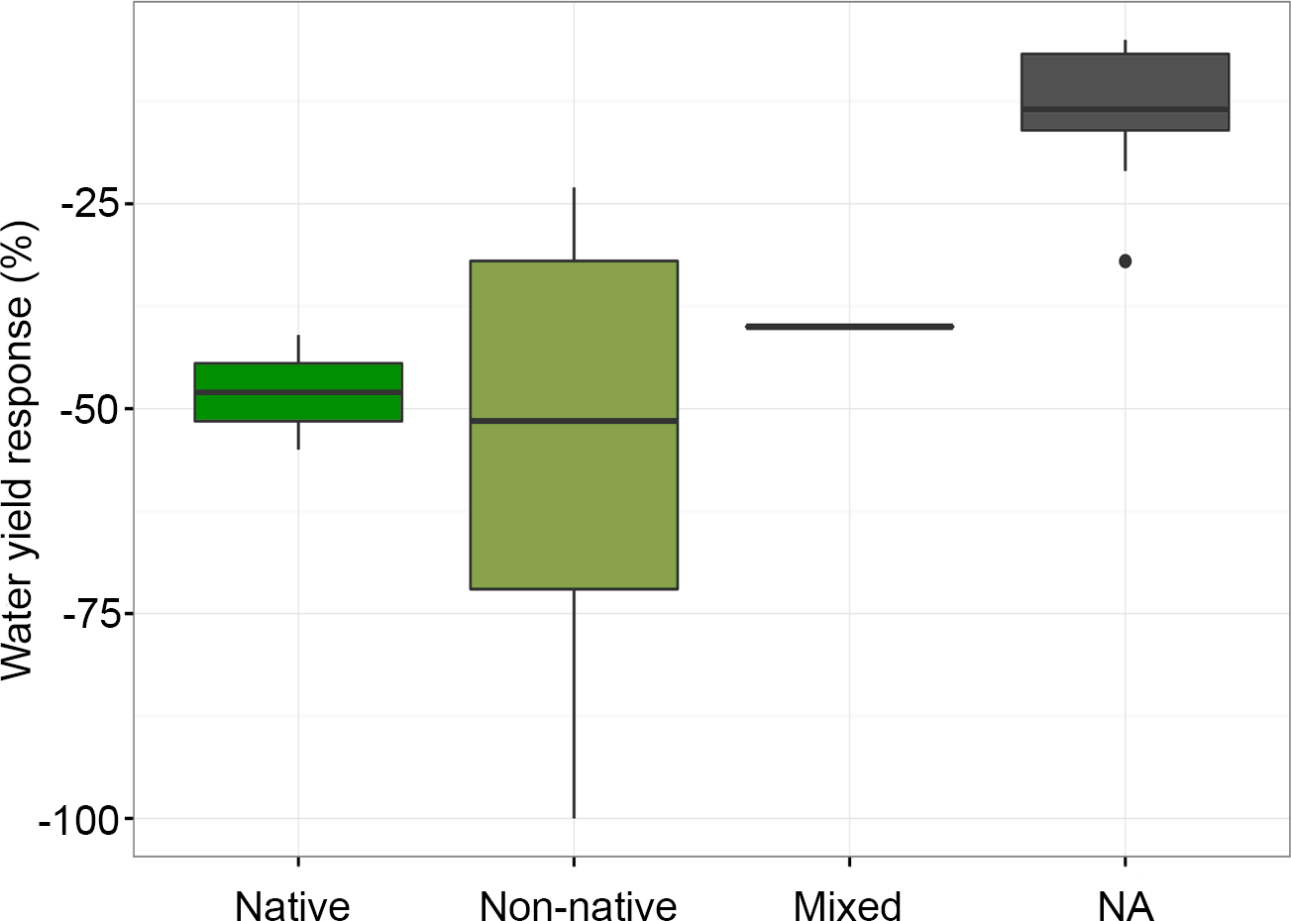
Magnitude of water yield change by forest type. Box plot showing the median, quartiles, maximum and minimum, and outlier values for changes in water yield following forest restoration or cover expansion. These results are based on a subset of case studies selected for meta-analysis (*n* = 37). NA refers to the category of studies with no information on tree species or forest types.

The magnitude of water yield reduction reported in studies with native, non-native, and mixed forest types varied significantly (*F* = 9.76; *p* < 0.001) (Fig 7). However, only the NA-non-native comparison is statistically significant, according to pairwise comparisons of group means.

**Fig 8:**
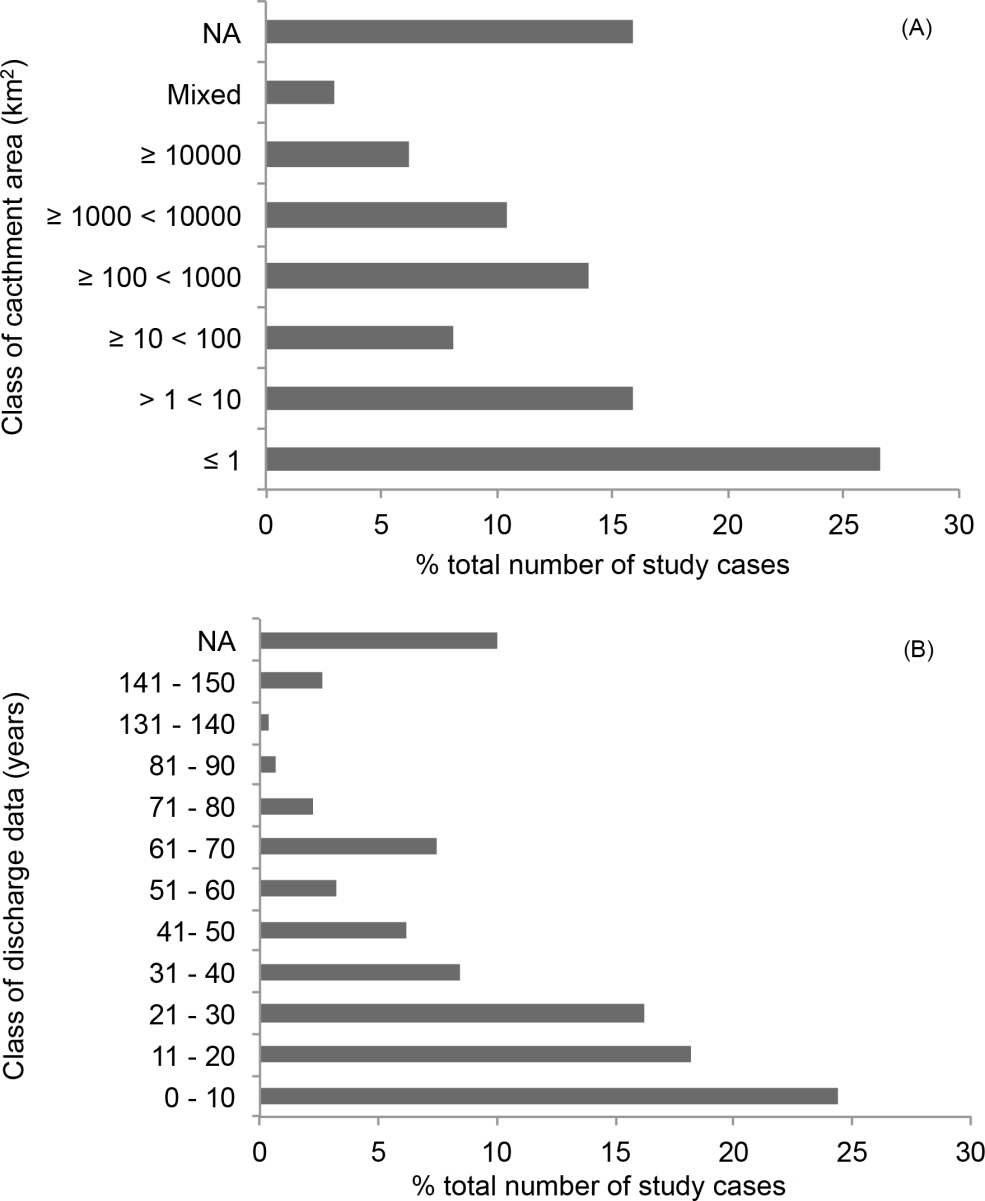
Distribution of studies by spatial and temporal scale categories. Percent distribution of study cases according to catchment area (a) and time scale of water yield dataset (b). The case studies included reported on water yield results from direct measurements.

Most of the water yield data available in the literature are from relatively small catchments (≤ 10 km^2^) and are relatively short-term (< 10 y) (Fig 8). The two smallest size categories (< 10 km^2^) include more than 50% of the study cases, while the two largest size categories include < 25% of the total number of studies (Fig 8A). Similarly, the majority of studies provided water yield with < 10 years of data while long-term data sets are relatively rare (Fig 8B).

**Fig 9:**
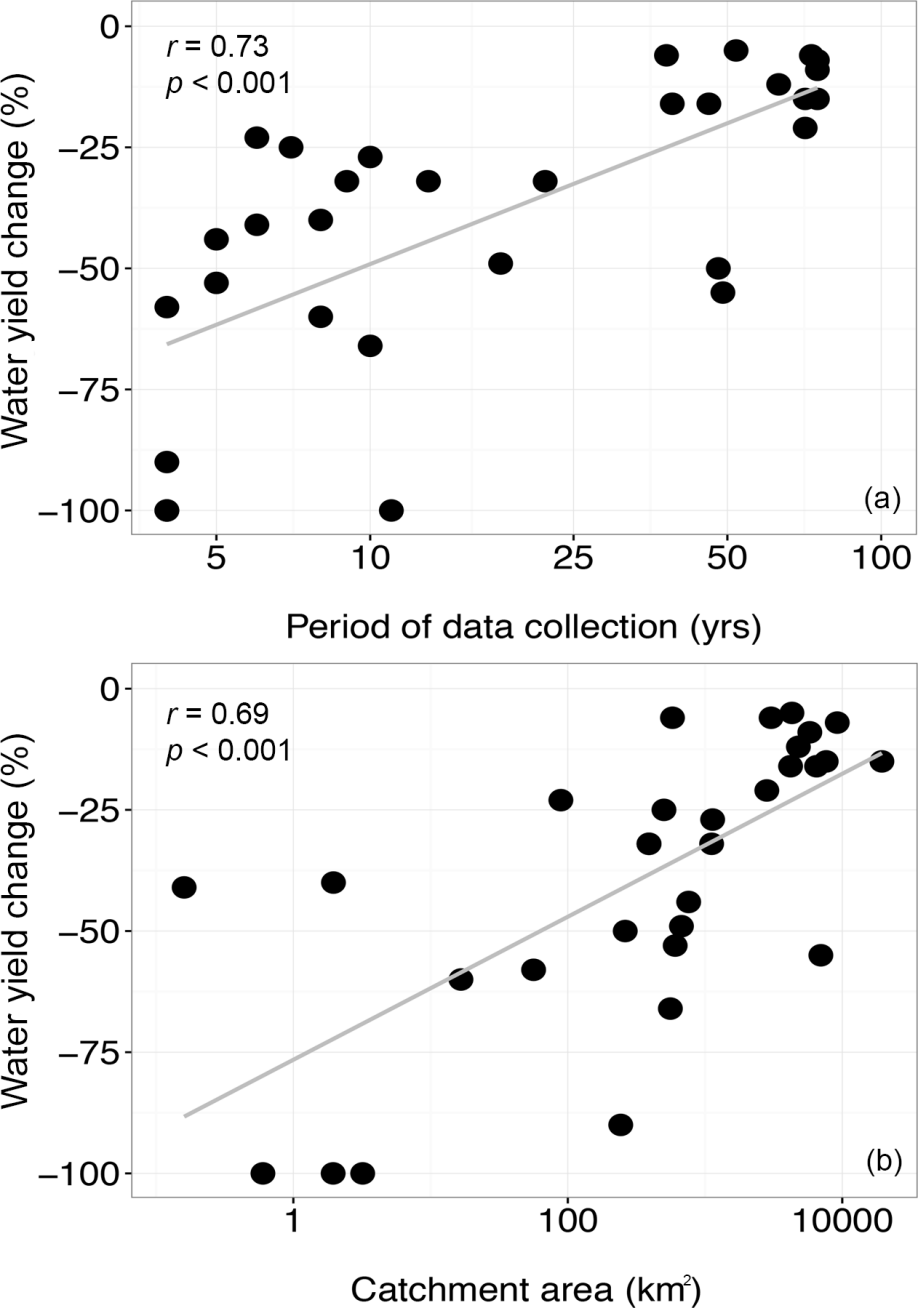
Meta-analyses of water yield response versus temporal and spatial scale of studies. Scatter plots with percentage change (decline) in water yield versus period of data collection (a) and catchment size in km^2^ (b). The data included are from a subset of study cases selected for meta-analyses, and the x-axis are log10 scaled. Spearman correlation results are indicated for both datasets.

The impacts of study duration and catchment size on water yield responses were examined using data from the subset of studies selected for meta-analysis (S2 Table). The magnitude of water yield reduction (calculated as the difference in water yields between pre- and post-forest restoration or forest cover expansion) in these studies was correlated with the number of years of the dataset (Fig 9A) and with the size of the catchment (Fig 9B). In both cases, the larger the temporal or spatial scale, the smaller the decrease in water yield. None of the studies in the subset used in the meta-analysis presented positive values, which means that no water yield gains were observed in any of these study cases.

**Fig 10:**
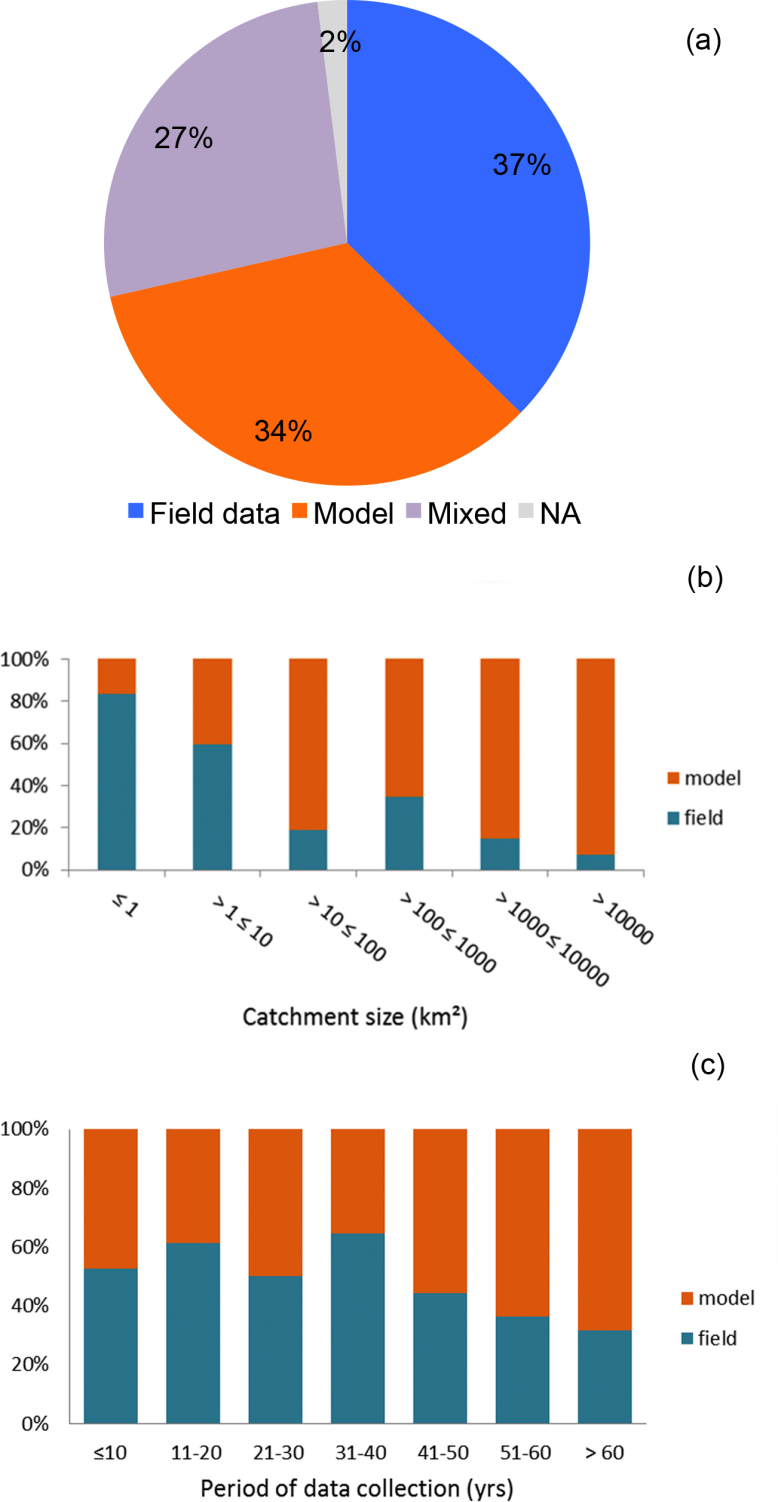
Studies with field and model water yield data. Percent distribution of study cases with data collected with different methods (a), and relative abundance of studies with field or model data according to spatial (b) and temporal (c) scales.

An equal number of studies selected in the review had water yield data from field measurements, models, or from a mixture of the two methods (Fig 10A). However, the dominant method of data acquisition varied according to the spatial and temporal scales of the study. While field data were predominant in small-catchment studies, modeled data were predominant in large-catchment ones (Fig 10B). Field data were also more abundant in studies with short-term datasets than in studies with long-term datasets, but differences were less pronounced among time-scale categories (Fig 10C).

**Fig 11:**
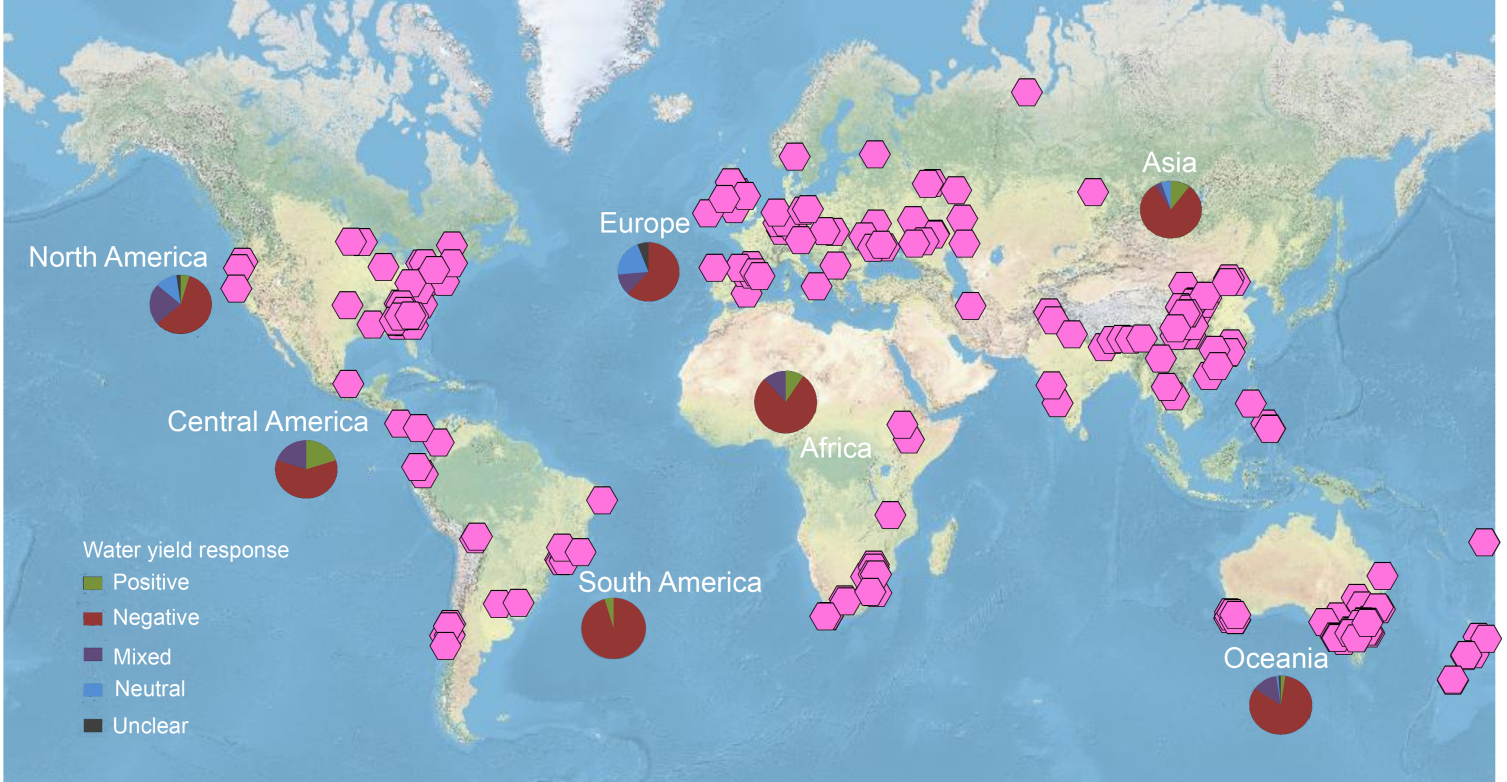
Geographic location of study cases and their water yield outcomes. Global distribution of study cases providing data on changes in water yield following forest restoration or forest cover expansion. The pie charts indicate the distribution of water yield responses reported in the studies from the different regions. Red represents a negative response, green a positive response, and purple mixed results. Neutral response is represented by light blue. Source for the world map is the US National Park Service (Natural Earth physical map; https://www.arcgis.com/home/item.html?id=c4ec722a1cd34cf0a23904aadf8923a0).

The geographic representativeness of the studies available in the literature and selected in the review was reasonable, with studies in all of the main continental regions of the world (Fig 11). Asia (including the Middle East) had the highest number of studies, followed by Oceania, North America, Africa, Europe, and South and Central Americas (Table 3). When normalized by the total land area of each continental region, Oceania emerged as the region with the highest density of studies, mostly due to the high number of studies in Australia, while Central and South Americas had the lowest density of studies (Table 3). The number of studies in Africa was relatively low as well.

**Table 3:** Number of study cases in each continental region. The number of cases are presented as absolute numbers and also normalized by the land area of the respective continent.

Continent	Total land area (10^6^ km^2^)	Number of case studies	Number of case studies per area
Asia + Middle East	44.579	88	1.9
Oceania	7.687	79	10.3
North America	24.256	43	1.8
Africa	30.065	41	1.4
South + Central America	18.363	23	1.3
Europe	9.938	32	3.2
TOTAL		308	

**Fig 12:**
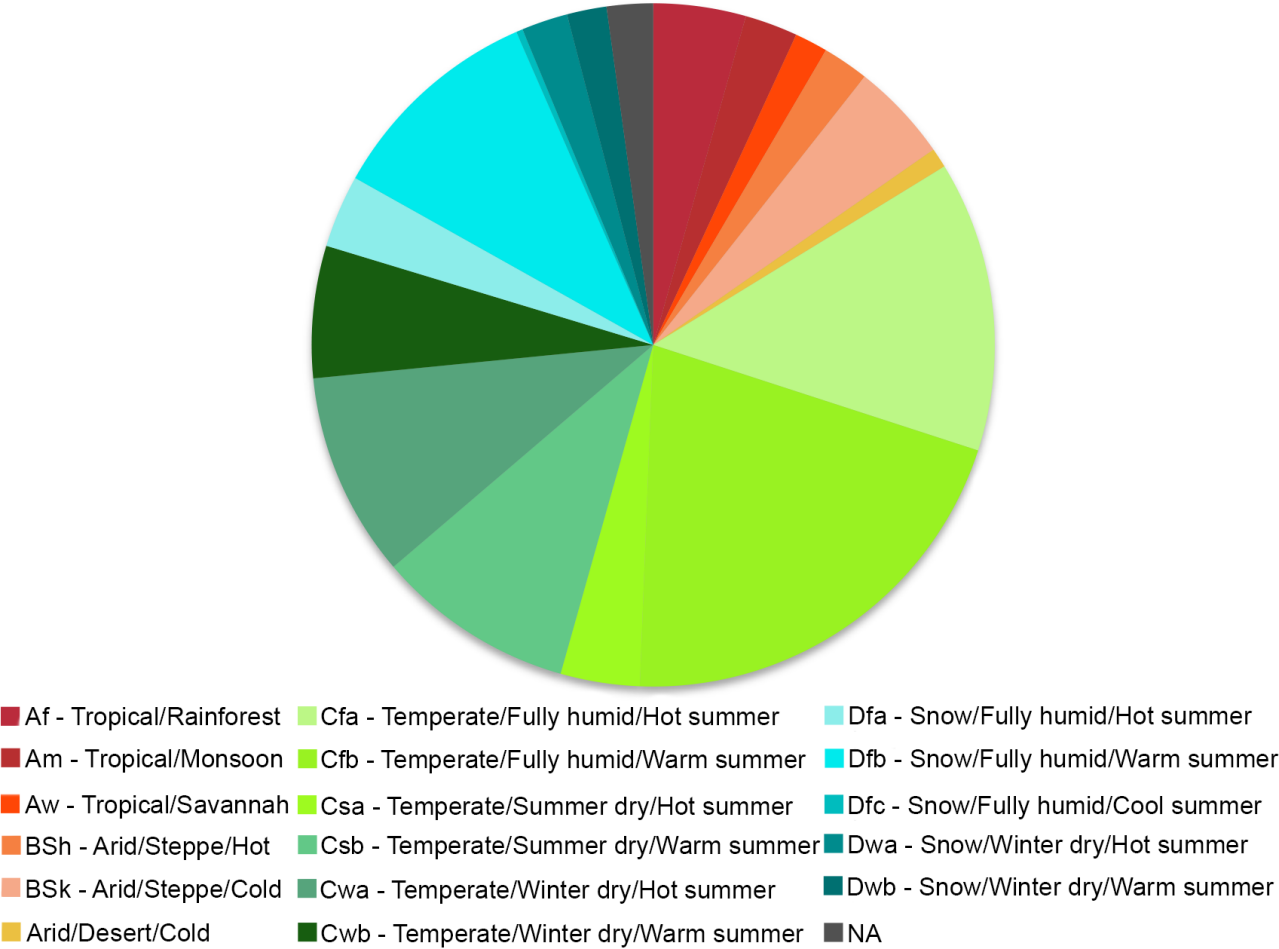
Study cases per Köppen-Geiger climate class. Percent distribution of study cases according to climate classes using the Köppen-Geiger classification system. The main climate groups include: (A) tropical, (B) dry arid and semiarid, (C) mild temperate, (D) continental, and (E) polar/alpine. The subgroups are divided according to average annual and monthly temperatures, total precipitation and precipitation seasonality.

The majority of studies were located in areas characterized as mild temperate oceanic (also known as marine or maritime) or humid subtropical, according to the Köppen-Geiger classification (Fig 12). Nevertheless, most of the other climate categories were represented by the studies selected in the review.

## Discussion

The primary goal of this systematic review was to provide a state-of-the-art synthesis of current evidence for the effects of forest restoration on water yields. As many researchers who have drawn inferences about the value of forest restoration for recovering water ecosystem services have studied diverse types of efforts to restore or expand forest cover, we included all of these types in our review (Fig 2). By analyzing the outcomes from these different types of efforts as well as differences in scale, methodology, and geographic location of studies available, we were able to identify potential sources of bias in the conclusions one might reach when reading the literature on water outcomes and forest restoration. This allowed us to identify knowledge gaps and quantitatively evaluate whether or not existing information can be extrapolated across different settings and regions of the world.

The great predominance of studies reporting reduced water yields following forest cover restoration or expansion in the review results corroborates the conclusions from some earlier assessments (e.g., [13,25,45]). This suggests that the implementation of forest restoration projects may have unintended negative impacts on the availability of water for human use. The mechanism believed to lead to reduced water yields following forest cover expansion is an alteration of the balance between rainfall, evaporation, and the runoff response of the system (e.g. [8,13,16,17,24,45]). Higher evapotranspiration, reduced water availability in soils and groundwater, and changes in energy fluxes (e.g., albedo, sensible, and latent heat) are some of the main mechanisms driving these changes [46]. However, as we discuss more fully below, the published studies available and selected in the review were mostly short-term and the forest restoration projects small in scale, which could limit the ability of researchers to adequately evaluate the potential for these projects to lead to positive water yield outcomes. If recovery of hydrological processes (e.g., increased soil infiltration), as some studies reported, is an indication of eventual recovery of water yield then there may be positive benefits in time.

### Potential obstacles to the recovery of surface water yields with forest cover restoration

Forests and the hydrological cycle are closely interlinked in the sense that while forests influence water flows and storage, their growth depends precisely on these processes. The influence of forests on the hydrological cycle varies across time and space, producing a complex dynamic system that is difficult to reproduce. In essence, the recovery of surface water yields depends on reinstating some key hydrological processes such as soil infiltration, groundwater recharge, and subsurface and groundwater flows [47,48,49], which depend on multiple interacting factors. Scale of the forest cover restoration, position of the restoration on the landscape, climate, local geology, and functional and structural complexity of the vegetation are some of the potential factors [50].

In some cases, such as in plantation forests and forestry systems, key processes such as soil infiltration and infiltration capacity may never improve due to the absence of natural understory vegetation or because of management activities, such as site preparation, cultivation, drainage, road construction, and logging that prevent the recovery of soil characteristics [14]. In some regions, such as the tropics, the recovery of infiltration capacity may be extremely difficult to achieve without soil restoration efforts because these soils tend to be particularly vulnerable to compaction and other structural changes [32]. Recognizing such constraints can facilitate the development of methods that can accelerate the recovery of hydrological processes in forest restoration, even on highly degraded landscapes [51].

There are more extreme cases, however, where a regime shift [52] prevents the recovery of key hydrological processes following deforestation. Depending on the condition of the deforested area prior to restoration, the positive feedbacks between the forest vegetation and its physical environment that are needed to push the system back into a stable forested state [53,54] may never be generated. In intensively farmed lands (which are commonly targeted for forest restoration), for instance, key hydrological processes may not be reversed through forest regrowth or reforestation if the land change and use resulted in soil degradation and infiltration and water storage capacity losses [55,56]. After a prolonged period without forest cover, some landscapes become unable to recover spontaneously and remain locked in a degraded state [46]. Agricultural pressure at the landscape scale can add another level of complexity to the problem [57].

### Characteristics of studies that may bias the review results

There are many possible biophysical reasons why water yield may not recover following forest restoration; however, the overwhelming predominance of studies in our review reporting decreases in water yields following forest cover expansion may also be related to the nature of the studies published to date. Most of these studies were completed on forestry systems or as part of reforestation or afforestation projects implemented without water benefits in mind (Fig 2). Afforestation projects, especially, are often located in areas unfavorable to water provision services (e.g., areas susceptible to prolonged droughts) and many of them are implemented for different reasons, such as to increase carbon sequestration or erosion control. Some of these involve exotic tree species, which are typically fast-growing and may not have the most efficient water use for local conditions (e.g., [38]). Even if native species were involved, changes in forest composition can lead to higher water use and consequent reduction in water yields [58]. Factors, such as the eco-hydrological setting [59,60], original land cover [59], and climate [61] are influential as well. However, the fact that there was a relatively limited number of studies involving forest restoration with native species (Figs 6 and 7), and probably fewer still where species composition of the restored forest was comparable to that of the original forest could have considerably limited the number of studies with positive water yield outcomes available in the literature and, consequently, selected in the review.

Further, most of the literature contained water yield datasets of 10 years or less, which is a relatively short period of time for forest development. Forest hydrology research has shown that, while water yields tend to increase immediately after deforestation, they usually decline during forest regrowth [62,63], sometimes to levels below the pre-deforestation period (e.g., [64]). When forests mature, water yields can reach a new equilibrium [45] and potentially increase to return to pre-deforestation levels, but this can take more than a decade. Consequently, except for studies that use the space-for-time design, short-term water yield data may be more likely to show negative results than long-term datasets.

The results of our meta-analysis clearly show an inverse relationship between magnitude of water yield reduction and length of the study dataset (Fig 9A). This suggests that water yields may recover over time and support the argument that the predominance of short-term studies may have created a bias towards a higher frequency of studies with negative water yield results in the review. However, it is important to note that studies that focused on longer temporal scales also tended to focus on larger catchments (e.g., > 100 km^2^) and use modeling approaches to evaluate hydrologic response (Fig 10B and 10C). Short-term studies, on the other hand, were usually done in relatively small catchments that were typically < 10 km^2^ in size. Therefore, the explicit influence of study length on the outcome of the selected studies is not clear-cut.

Catchment size can be relevant to the outcome of the results if the scale of forest expansion alters precipitation patterns through climatic feedbacks, as suggested by [17]. Despite the lack of empirical evidence to date indicating large-scale forest cover expansion increases precipitation and channel runoff through climatic feedbacks, new studies are showing evidence that deforestation in the Amazon affects regional changes in climate and the water cycle [65]. This can be used as an analogue for possible effects of large-scale forest restoration on regional climate.

Our meta-analysis showed a significant inverse correlation between the magnitude of water yield reduction and catchment size, suggesting that spatial scale was influential (Fig 9B). Unfortunately, however, the catchment sizes reported in studies did not necessarily reflect the size of the forest intervention, making it difficult to determine if the scale of the intervention was driving factor determining changes in water yields or if the results were an artifact of differences in the percentage of restored forest cover within the catchments of the studies selected. If studies involving larger catchments had relatively small areas of restored forest cover in comparison to small-catchment studies, then the magnitude of water yield changes was driven by the relative size of the intervention rather than the absolute size. Such information can be also be valuable to decision makers interested in preventing a major decline in water yields associated with forest restoration.

### Geographic and climate representativeness of studies

While our synthesis included a large number of studies that spanned more than 50 years and included all of the major geographic continental regions of the world (Fig 11), their geographic distribution is quite uneven (Table 3). Most studies were from Oceania and particularly from Australia. Many of the latter were related to efforts to purposely reduce water quantity and groundwater recharge to reverse dryland salinization in certain regions of the country [25,66,67], which potentially could have increased the prevalence of negative versus positive water yield results in the review.

When normalized by total continental area, the number of studies from Oceania was still relatively high, but Europe emerged as the region with the second largest number of studies per unit area to date. The number of studies per area in Asia is low, yet, studies from this region make up nearly a third of all the studies selected, and thus, like Oceania, play a large role in the patterns that emerged from our review. In contrast, the total and per unit area number of studies from South America is low despite the growing number of reforestation and forest restoration projects implemented in these fast-changing region, many of which are meant to address water shortages and prevent water insecurity [68–71].

Our climate representativeness analysis revealed that, despite the large number of studies from Australia, which is known for its vast arid lands and water scarcity issues [72], the predominant climate type represented in studies available in the literature to date is temperate. Studies from the humid tropics and subtropics are lacking in Australia and elsewhere, but are available for Central America. Interestingly, the highest percentage of studies reporting positive water yields was in humid tropical regions of Central America (Fig 11). If this holds true for other humid tropical regions, then South American regions where water shortages and deforestation are motivating forest restoration projects may find more positive outcomes than the overall results of this review suggest.

### Other potential hydrological benefits of forest cover restoration

The results of the review suggest that, in most cases, forest restoration will likely lead to a decrease in annual water yields, baseflow and groundwater, at least temporarily. However, other positive hydrological outcomes such as increased soil infiltration and reduced peak flows and flood frequency were predominant (Fig 5). This suggests that even if forest restoration projects do not yield positive benefits for people and biodiversity in the form of blue water availability, they should help with the provision of green water, erosion control and regulation of sediment and nutrient transport in waterways (e.g., [73]). It is important to acknowledge, though, that the recovery of these services may be protracted since a myriad of factors such as soil use history, forest intervention type, and vegetation type [19,32,33] can affect surface and subsurface conditions and strongly influence recovery.

Increased soil infiltration is a particularly important outcome from forest cover restoration because it can lead to higher groundwater recharge [48,74], depending on rates of plant growth and ET from forest plants. Unfortunately, the effect of terrestrial plant restoration on groundwater storage is complex and difficult to quantify, and predicting changes in groundwater recharge based on infiltration rates can result in large errors. In contrast, decreased peak flows and flood magnitude or frequency is relatively easy to detect and can serve as a clear indicator of the hydrologic benefits of forest restoration. Nevertheless, while such reductions are assumed to be a positive outcome, the positive connotation depends on the perspective of the subject affected by floods, as discussed in depth by [14]. Hence, the benefits of peak flow reduction and flood control should be carefully evaluated for each individual project. Yet, reduction of peak flows can improve the biodiversity and ecological integrity of waterways draining developed watersheds [75,76], and indirectly affect the well-being of people. This is particularly true for small streams, which are more vulnerable to the impacts of land cover changes [77].

## Conclusions

Systematic reviews are considered efficient methods of providing unbiased, science-based information to guide decision-making [35]. While this study reinforces the conclusions some other authors have reached (i.e., that water yields are likely to decrease with forest cover restoration), it has uncovered a tremendous bias in the portfolio of studies available to date–most studies are short-term, conducted in relatively small catchments, and focused on forestry and exotic species. Further, few projects have been explicitly designed to examine major factors that can influence the hydrological response and water yield outcome of forest restoration. This limits our capacity to generalize and predict changes in water yields as a function of geographic region, methods implemented, scale of the project, and time since implementation of the project. Evidence of recovery of key hydrological processes, such as soil infiltration and flood regime in some studies, suggest that water yields and channel runoff may eventually reach pre-deforestation levels after forest cover restoration, but this may take considerable time.

Studies are particularly lacking in the humid tropics, an area targeted to have a growing number of forest restoration projects implemented over the next several decades for climate change mitigation and other purposes. Central America has the highest percentage of positive water yield results from forest cover expansion, but this is based on only 5 studies to date, making it difficult to extrapolate the findings to other regions of the humid tropics. Studies in which empirical data are collected on large-scale restoration projects are also lacking. Our review did find that studies using primarily modeling approaches found smaller decreases in the magnitude of water yields with catchment size. However, because these studies did not generally report the actual scale of the restoration, this relationship may simply reflect the fact that the amount of forest cover expansion in large catchments was relatively small, resulting in relatively small changes in water yields. While the water outcomes were still negative in large catchments, the implication that restoring larger areas may result in better outcomes points toward a strong need for well-monitored large scale projects.

Considering the socioeconomic consequences of unintended changes in water yields to local and downstream communities, it is obvious that many knowledge gaps need to be addressed in order to improve our capacity to plan restoration projects. We need to advance our understanding of how forest cover restoration affects the hydrology of the landscape at each stage of the process in order to better predict outcomes and implement adaptive management. Undoubtedly, outcomes will vary spatially and temporally, so targeted areas and expectations must be adjusted accordingly. Hopefully, as restoration methods and strategies evolve, restored forests age, and more large-scale restoration projects are implemented, such knowledge limitations will diminish. The collection of pre- and post-restoration data for new restoration projects and programs could significantly enhance our basic understanding of the hydrological responses of forest restoration. Meanwhile, recognizing the weaknesses of our present understanding on the effects of forest restoration on water can help prevent unintended consequences and improve positive outcomes.

## Supporting information

S1 Table Selected papers. Complete list of publications selected in the systematic review and summary of the relevant information used in analyses. Publications with more than one study case had the information entered separately for each one.
(PDF)

S2 Table Database for meta-analises. Summary of data extracted from a sub-set of publications for the meta-analyses. The paper ID column corresponds to the same column in S1 Table.
(PDF)

S1 File PRISMA checklist. Checklist of Preferred Reporting Items for Systematic Reviews and Meta-Analyses (PRISMA).
(DOC)
