# Supplementary material for: Impacts of forest restoration on water yield: A systematic review
Source: PLoS One. 2017 Aug 17;12(8):e0183210. doi: 10.1371/journal.pone.0183210 (PMC5560669; doi:10.1371/journal.pone.0183210)
Supplement: S1 Table — Complete list of publications selected in the systematic review and summary of the relevant information used in analyses. Publications with more than one study case had the information entered separately for each one. (PDF) [file pone.0183210.s001.pdf]

Table S1: Database

Paper ID	Publication Reference				Study Information			Coordinates		Type of Intervention						Direct Metric		Direct Annual Water Yield Response				Baseflow			Indirect Water Yield Metrics									Forest Type				Data Acquisition Method				Continent					
	Author(s)	Publication name	Publication year	Publication title	Study case name	Years of discharge data	Catchment area (km^2)	Latitude	Longitude	Forest Restoration (explicit)	Forest Restoration (implicit)	Forestry	Afforestation/Forestation	Reforestation	Forest Regrowth	Water yield	Stream flow	Positive	Negative	No change	Unclear	Positive	Negative	Neutral	Increased Flooding/ Peak Flow	Decreased Flooding/ Peak Flow	Neutral Flooding/ Peak Flow	Increased GW level/ recharge	Decreased GW level/ recharge	Neutral GW level/ recharge	Increased soil infiltration / capacity	Decreased soil infiltration / capacity	Neutral soil infiltration / capacity	Native Forest	Non-native Forest	Mixed	ND Species	Field-Data	Model	Mixed	Historical Data/ Meta-data	Africa	Asia+ Middle East	Oceania	South+ Central America	Europe	North America
1	Andréassian et al.	Water Resources Research	2003	A distribution-free test to detect gradual changes in watershed behavior	And_2003_NAEW	29	0.18	40.36	-81.79	1	0	0	0	1	0	1	0	0	0	1	0	0	0	1	0	0	0	0	0	0	0	0	0	0	1	0	0	1	0	0	0	0	0	0	0	0	1
2	Arifeen & Chaudhry	The Pakistan Journal of Forestry	1997	Effect of vegetation and engineering control structures on surface runoff and sediment yield at Fizagar, Swat	Ari_1997_Catch_1	9	0.20	34.75	72.37	1	1	0	0	1	0	1	1	0	1	0	0	0	0	0	0	0	0	0	0	0	0	0	0	1	1	1	0	1	0	0	0	0	1	0	0	0	0
					Ari_1997_Catch_2	9	0.13	34.75	72.37	1	1	0	1	0	0	1	1	0	1	0	0	0	0	0	0	0	0	0	0	0	0	0	0	1	1	1	0	1	0	0	0	0	1	0	0	0	0
					Ari_1997_Catch_3	9	0.13	34.75	72.37	0	0	0	0	1	0	1	1	0	1	0	0	0	0	0	0	0	0	0	0	0	0	0	0	1	1	1	0	1	0	0	0	0	1	0	0	0	0
					Ari_1997_Catch_4	9	0.04	34.75	72.37	0	0	0	0	1	0	1	1	0	1	0	0	0	0	0	0	0	0	0	0	0	0	0	0	1	1	1	0	1	0	0	0	0	1	0	0	0	0
3	Banks	Forestry in South Africa	1961	The hydrological effects of riparian and adjoining vegetation	Ban_1961_Bos	40	NA	-34.00	19.00	0	0	0	0	0	0	1	0	0	1	0	0	0	0	0	0	0	1	0	0	0	0	0	0	0	1	0	0	1	0	0	0	1	0	0	0	0	0
					Ban_1961_Bie	40	NA	-34.00	19.00	0	0	0	0	0	0	1	0	0	1	0	0	0	0	0	0	0	1	0	0	0	0	0	0	0	1	0	0	1	0	0	0	1	0	0	0	0	0
					Ban_1961_Tie	40	NA	-34.00	19.00	0	0	0	0	0	0	1	0	1	0	0	0	0	0	0	0	0	1	0	0	0	0	0	0	0	1	0	0	1	0	0	0	1	0	0	0	0	0
					Ban_1961_LAN	40	NA	-34.00	19.00	0	0	0	0	0	0	1	0	1	0	0	0	0	0	0	0	0	1	0	0	0	0	0	0	0	1	0	0	1	0	0	0	1	0	0	0	0	0
4	Bari et al.	19th International Congress on Modelling and Simulation, Perth, Australia, 12-16 December 2011	2011	The impact of climate change and plantation developent on streamflow in the Denmark River catchment, Western Australia	Bar_2011_Lin	65	500.00	-34.80	117.28	0	1	1	0	1	1	1	0	0	1	1	0	0	0	0	0	0	0	0	1	0	0	0	0	0	0	0	1	0	1	0	0	0	0	1	0	0	0
5	Bari & Schofield	Journal of Hydrology	1992	Lowering of a shallow, saline water table by extensive eucalypt reforestation	Bar_1992_Wel_1	10	2.84	-33.20	116.02	0	1	0	0	1	0	1	0	0	1	0	0	0	0	0	0	0	0	0	0	0	0	0	0	1	1	1	0	1	0	0	0	0	0	1	0	0	0
6	Begueria et al.	Ambio	2003	Assessing the effect of climate oscillations and land-use changes on streamflow in the Central Spanish Pyrenees	Beg_2003_Catch	50	74.30	43.61	0.42	0	1	0	0	0	1	1	0	0	1	0	0	0	0	0	0	0	0	0	0	0	0	0	0	1	0	0	0	0	1	0	0	0	0	0	0	1	0
7	Bellot et al.	Landscape and Urban Planning	2001	Likely effect of land use changes on the runoff and aquifer recharge in a semiarid landscape using a hydrological model	Bel_2001_Ven	20	6.10	38.47	-0.62	0	1	0	1	0	1	1	0	1	1	0	0	0	0	0	0	0	0	0	1	0	0	0	0	1	0	0	0	0	1	0	0	0	0	0	0	1	0
8	Bellot & Chirino	Ecological Modelling	2013	Hydrobal: An eco-hydrological modelling approach for assessing water balances in different vegetatoin types in semi-arid areas	Bel_2013_Ven	3	16.00	38.47	-0.62	0	1	0	1	0	1	0	0	1	1	0	0	0	0	0	0	0	0	0	1	0	0	0	0	0	1	0	0	0	1	0	0	0	0	0	0	1	0
9	Bi et al.	The Forestry Chronicle	2014	Scale effects and variability of forest water yield relationships on the Loess Plateau, China	Bi_2014_SYN	54	632,520.00	38.01	110.18	0	1	0	0	0	1	1	0	0	1	0	0	0	0	0	0	0	0	0	0	0	0	0	0	0	0	0	1	1	0	0	0	0	1	0	0	0	0
10	Birkel et al.	Hydrological Sciences Journal	2012	Modelling the impacts of land-cover change on streamflow dynamics of a tropical rainforest headwater catchment	Bir_2012_Sar_1	9	73.40	10.28	-84.19	0	1	0	0	1	0	1	0	0	1	0	0	0	0	0	0	0	0	0	0	0	0	0	0	1	0	0	0	0	1	0	0	0	0	0	1	0	0
					Bir_2012_Sar_3	9	73.40	10.28	-84.19	0	1	0	0	1	0	1	0	0	1	0	0	0	0	0	0	0	0	0	0	0	1	0	0	1	0	0	0	0	1	0	0	0	0	0	1	0	0
11†	Birkinshaw et al.	Journal of Hydrology	2014	45 years of non-stationary hydrology over a forest plantation growth cycle, Coalburn catchment, Northern England	Bir_2014_Coa	44	1.50	55.04	-2.57	0	0	1	1	0	0	1	0	0	1	0	0	0	0	0	0	1	0	0	0	0	0	0	0	0	1	0	0	0	1	0	0	0	0	0	0	1	0
12†	Borg et al.	Journal of Hydrology	1988	Streamflow and stream salinity in a small water supply catchment in southwest western Australia after reforestation	Bor_1988_Pad	8	1.96	-33.81	116.01	1	1	0	0	0	1	1	0	0	1	0	0	0	0	0	0	0	0	0	0	0	0	0	0	1	1	1	0	1	0	0	0	0	0	1	0	0	0
13	Borg et al.	Journal of Hydrology	1988	The effect of logging and regeneration on groundwater, streamflow and stream salinity in the southern forest of Western Australia	Bor_1988_Cro	10	1.14	-34.42	116.35	1	1	0	0	0	1	1	0	0	1	0	0	0	0	0	0	0	0	0	0	0	0	0	0	1	0	0	0	1	0	0	0	0	0	1	0	0	0
					Bor_1988_Poo	10	1.21	-34.42	115.81	1	1	0	0	0	1	1	0	0	1	0	0	0	0	0	0	0	0	0	0	0	0	0	0	1	0	0	0	1	0	0	0	0	0	1	0	0	0
					Bor_1988_Iff	10	1.14	-34.56	115.95	1	1	0	0	0	1	1	0	0	1	0	0	0	0	0	0	0	0	0	0	0	0	0	0	1	0	0	0	1	0	0	0	0	0	1	0	0	0
					Bor_1988_Moo	10	1.12	-34.56	115.96	1	1	0	0	0	1	1	0	0	1	0	0	0	0	0	0	0	0	0	0	0	0	0	0	1	0	0	0	1	0	0	0	0	0	1	0	0	0
14	Bosch	South African Forestry Journal	1979	Treatment effects on annual and dry period streamflow at Cathedral Peak	Bos_1979_CII	26	1.90	-29.00	29.25	1	1	0	0	0	1	1	0	0	1	1	0	0	0	0	0	0	0	0	0	0	0	0	0	0	1	0	0	1	0	0	0	1	0	0	0	0	0
					Bos_1979_CIII	22	1.42	-29.00	29.25	1	1	0	0	0	1	1	0	0	1	1	0	0	0	0	0	0	0	0	0	0	0	0	0	0	1	0	0	1	0	0	0	1	0	0	0	0	0
15	Bos_1982_REV	Journal of Hydrology	1982	A review of catchment experiments to determine the effect of vegetation changes on water yield and evapotranspiration	Bos_1982_REV	15	0.01 - 146.00	NA	NA	0	0	1	1	0	1	1	0	0	1	0	0	0	0	0	0	0	0	0	0	0	0	0	0	1	1	0	0	1	0	0	1	0	0	0	0	0	1
16	Bren & Hopmans	Journal of Hydrology	2007	Paired catchments observations on the water yield of mature eucalypt and immature radiata pine plantations in Victoria, Australia	Bre_2007_Cle	31	0.46	-36.70	146.82	0	0	1	1	0	0	1	0	1	0	0	0	0	0	0	0	1	0	0	0	0	0	0	0	0	1	0	0	1	0	0	0	0	0	1	0	0	0
17	Brown et al.	Journal of Hydrology	2013	Impact of forest cover changes on annual streamflow and flow duration curves	Bro_2013_SA	57	0.26 - 2.00	-30.55	23.00	0	0	1	1	0	0	1	0	0	1	0	0	0	0	0	0	0	0	0	0	0	0	0	0	0	1	0	0	0	0	0	1	1	0	0	0	0	0
					Bro_2013_Red	4	1.95	-35.27	148.25	0	0	1	1	0	0	1	0	0	1	0	0	0	0	0	0	0	0	0	0	0	0	0	0	0	1	0	0	0	0	0	1	0	0	1	0	0	0
					Bro_2013_Wai	9	2.18	-45.83	169.90	0	0	1	1	0	0	1	0	0	1	0	0	0	0	0	0	0	0	0	0	0	0	0	0	0	1	0	0	0	0	0	1	0	0	1	0	0	0
18	Burguete et al.	Natural Hazards and Earth System Sciences	2002	Numerical simulation of runoff from extreme rainfall events in a mountain water catchment	Bur_2002_Ebr	NA	NA	42.23	-1.11	0	0	0	0	1	0	1	0	0	0	0	1	0	0	0	0	1	0	0	0	0	0	0	0	0	0	0	1	0	1	0	0	0	0	0	0	1	0
19	Buttle	Hydrological Processes	2011	Streamflow response to headwater reforestation in the Ganaraska River Basin, southern Ontario, Canada	But_2011_GRB	62	267.00	43.95	-78.29	0	1	1	0	1	0	1	0	0	1	0	0	1	0	1	0	1	0	0	0	0	0	0	0	0	1	0	0	1	0	0	0	0	0	0	0	0	1
					But_2011_DCB	62	249.00	43.84	-79.09	0	0	1	0	0	0	1	0	0	0	1	0	1	0	1	0	1	0	0	0	0	0	0	0	0	1	0	0	1	0	0	0	0	0	0	0	0	1
20†	Buttle	The Canadian Geographer	1994	Hyrological response to reforestation in the Ganaraska River Basin, southern Ontario	But_1994_Gan	48	262.00	43.95	-78.28	0	1	0	0	1	0	1	0	0	1	0	0	1	0	1	0	1	0	0	0	0	0	0	0	0	1	0	0	1	0	0	0	0	0	0	0	0	1
21	Cao et al.	Water Resources Management	2009	Modelling impacts of land cover change on critical water resources in the Motucka River catchment, New Zealand	Cao_2009_Mot	10	2,180.00	-41.38	172.95	0	0	0	1	1	0	1	0	0	1	0	0	0	1	0	0	0	0	0	0	0	0	0	0	0	1	0	0	0	1	0	0	0	0	1	0	0	0
22	Chandler	Forest Ecology and Management	2006	Reversibility of forest conversion impacts on water budgets in tropical karst terrain	Cha_2006_For	1	NA	10.30	124.80	0	1	0	0	0	1	1	0	0	1	0	0	1	0	1	0	0	0	1	0	0	1	0	0	1	0	0	0	0	1	0	0	0	1	0	0	0	0
23	Chappell & Tych	Hydrological Processes	2012	Identifying step changes in single streamflow and evaporation records due to forest cover change	Cha_2012_Upp	58	37.50	54.00	-2.60	0	0	1	1	0	0	1	0	0	1	0	0	0	0	0	0	0	0	0	0	0	0	0	0	0	1	0	0	0	1	0	0	0	0	0	0	1	0
24	Cornish & Vertessy	Journal of Hydrology	2001	Forest age-induced changes in evapotranspiration and water yield in a eucalypt forest	Cor_2001_Cor	22	0.42	-32.58	152.11	0	1	0	0	0	1	1	0	0	1	0	0	0	0	0	0	0	0	0	0	0	0	0	0	1	0	0	0	1	0	0	0	0	0	1	0	0	0
					Cor_2001_Jac	22	0.13	-32.58	152.11	0	1	0	0	0	1	1	0	0	1	0	0	0	0	0	0	0	0	0	0	0	0	0	0	1	0	0	0	1	0	0	0	0	0	1	0	0	0
					Cor_2001_Kok	22	0.97	-32.58	152.11	0	0	1	0	0	0	1	0	0	1	0	0	0	0	0	0	0	0	0	0	0	0	0	0	0	0	0	0	1	0	0	0	0	0	1	0	0	0
					Cor_2001_Coa	22	0.38	-32.58	152.11	0	0	1	0	0	0	1	0	0	1	0	0	0	0	0	0	0	0	0	0	0	0	0	0	0	0	0	0	1	0	0	0	0	0	1	0	0	0
25	Cornish	Journal of Hydrology	1993	The effects of logging and forest regeneration on water yields in a moist eucalypt forest in New South Wales, Australia	Cor_1993_Cor	12	0.42	-32.58	152.11	0	1	0	0	0	1	1	0	0	1	0	0	0	0	0	0	0	0	0	0	0	0	0	0	1	0	0	0	1	0	0	0	0	0	1	0	0	0
					Cor_1993_Jac	12	0.13	-32.58	152.11	0	1	0	0	0	1	1	0	0	1	0	0	0	0	0	0	0	0	0	0	0	0	0	0	1	0	0	0	1	0	0	0	0	0	1	0	0	0
					Cor_1993_Kok	12	0.97	-32.58	152.11	1	0	1	0	0	0	1	0	0	1	0	0	0	0	0	0	0	0	0	0	0	0	0	0	0	0	0	0	1	0	0	0	0	0	1	0	0	0
					Cor_1993_Coa	12	0.38	-32.58	152.11	1	0	1	0	0	0	1	0	0	1	0	0	0	0	0	0	0	0	0	0	0	0	0	0	0	0	0	0	1	0	0	0	0	0	1	0	0	0
26	D'Ippolito et al.	iForest - Biogeosciences and Forestry	2013	Reforestation and land use change in a drainage basin of southern Italy	D'Ip_2013_Cos	56	NA	39.80	16.27	0	1	0	0	1	0	1	0	0	0	1	0	0	0	0	0	0	0	0	0	0	0	0	0	1	0	0	0	1	0	0	0	0	0	0	0	1	0
27	Daño	Proceedings of the Fiji Symposium	1990	Effect of buring and reforestation on grassland watersheds in the Phillipines	Dan_1990_Catch	15	NA	14.94	121.02	0	1	0	0	1	0	1	0	0	1	0	0	0	0	0	0	0	0	0	0	0	0	0	0	0	1	0	0	1	1	1	0	0	1	0	0	0	0
28	Descheemaeker et al.	The Rangeland Journal	2009	Changes in water flows and water productivity upon vegetation regeneration on degraded hillslopes in northern Ethiopia: a water balance modelling exercise	Des_2009_Ade	20	NA	6.49	38.52	0	1	0	0	0	1	1	0	0	1	0	0	0	0	0	0	0	0	1	0	0	1	0	0	0	1	0	0	1	1	1	0	1	0	0	0	0	0
					Des_2009_Kun	20	NA	6.49	38.52	0	1	0	0	0	1	1	0	0	1	0	0	0	0	0	0	0	0	1	0	0	1	0	0	1	0	0	0	1	1	1	0	1	0	0	0	0	0
					Des_2009_May	20	NA	6.49	38.52	0	1	0	0	0	1	1	0	0	1	0	0	0	0	0	0	0	0	1	0	0	1	0	0	1	0	0	0	1	1	1	0	1	0	0	0	0	0
29	Duncan & Collins	Journal of Hydrology, New Zealand	2013	Simple estimation of the effects on runoff of afforestation under different rainfall regimes	Dun_2013_Tar	NA	906.00	-38.00	176.00	0	0	0	1	0	0	1	1	0	1	0	0	0	0	0	0	0	0	0	0	0	0	0	0	0	0	0	1	0	1	0	0	0	0	1	0	0	0
30	Dymond et al.	Journal of Environmental Management	2012	Tradeoffs between soil, water, and carbon - A national scale analysis from New Zealand	Dym_2012_NL	NA	NA	-41.28	174.77	0	1	1	0	1	0	1	0	0	1	0	0	0	0	0	0	0	0	0	0	0	0	0	0	0	1	0	0	0	1	0	0	0	0	1	0	0	0
31	Fahey & Watson	Journal of Hydrology, New Zealand	1991	Hydrological impacts of converting tussock grassland to pine plantation, Otago, New Zealand	Fah_1991_GH2	10	3.10	-45.83	169.90	1	1	0	0	1	0	1	0	0	1	0	0	0	0	0	0	1	0	0	0	0	0	0	0	0	1	0	0	1	0	0	0	0	0	1	0	0	0
32	Fahey et al.	Journal of Hydrology, New Zealand	2010	Using the WATYIELD water balance model to predict catchment water yields and low flows	Fah_2010_GH2	27	3.10	-45.83	169.90	1	1	1	0	1	0	1	0	0	1	0	0	0	1	0	0	0	0	0	0	0	0	0	0	0	0	0	1	0	1	0	0	0	0	1	0	0	0
33	Fahey & Jackson	Agricultural and Forest Meteorology	1997	Hydrological impacts of converting native forests and grasslands to pine plantations, South Island, New Zealand	Fah_1997_DC1	14	0.08	-41.60	173.00	0	0	1	1	1	0	1	0	0	1	0	0	0	1	0	0	1	0	0	0	0	0	0	0	0	1	0	0	1	0	0	0	0	0	1	0	0	0
					Fah_1997_DC4	14	0.20	-41.60	173.00	0	0	1	1	1	0	1	0	0	1	0	0	0	1	0	0	1	0	0	0	0	0	0	0	0	1	0	0	1	0	0	0	0	0	1	0	0	0
					Fah_1997_GH2	13	3.10	-45.83	169.90	0	0	1	1	0	0	1	0	0	1	0	0	0	1	0	0	1	0	0	0	0	0	0	0	0	1	0	0	1	0	0	0	0	0	1	0	0	0
34	Fahey et al.	*Environmental Forest Science*, in Forestry Sciences pp 395-404	1998	Hydrological effects of afforestation and pasture improvement in montane grasslands, South Island, New Zealand	Fah_1998_Wai	16	2.18	-45.83	169.90	0		1	0	0	0	1	0	0	1	0	0	0	1	0	0	0	0	0	0	0	0	0	0	0	1	0	0	0	1	0	0	0	0	1	0	0	0
35	Farley et al.	Global Change Biology	2005	Effects of afforestation on water yield: a global synthesis with implications for policy	Far_2005_SYN	NA	NA	NA	NA	0	0	0	0	1	0	1	0	0	1	0	0	0	1	0	0	0	0	0	0	0	0	0	0	0	1	0	0	0	1	0	1	0	0	0	0	0	0
36	Feng et al.	Hydrology and Earth System Sciences	2012	Regional effects of vegetation restoration on water yield across the Loess Plateau	Fen_2012_REG	8	632,520.00	36.90	107.52	1	0	0	1	0	0	1	0	0	1	0	0	0	0	0	0	0	0	0	0	0	0	0	0	0	0	0	1	0	1	0	0	0	1	0	0	0	0
37	Gallart & Llorens	Physics and Chemistry of the Earth	2004	Observations on land cover changes and water resources in the headwaters of the Ebro catchment, Iberian Peninsula	Gal_2004_REG	50	10,646.00	41.90	0.52	0	1	0	0	1	1	1	0	0	1	0	0	0	0	0	0	0	0	0	0	0	0	0	0	0	1	0	0	0	1	0	0	0	0	0	0	1	0
38	Ghimire et al.	Hydrology and Earth System Sciences	2014	Negative trade-off between changes in vegetation water use and infiltration recovery after reforesting degraded pasture land in the Nepalese Lesser Himalaya	Ghi_2014_Jik	0	111.40	27.62	85.55	0	1	0	0	1	0	1	0	0	1	0	0	0	1	0	0	0	0	0	0	0	0	0	1	0	1	0	0	1	0	0	0	0	1	0	0	0	0
39	Ghimire et al.	Journal of Geophysical Research: Earth Surface	2013	Reforesting severely degraded grassland in the Lesser Himalaya of Nepal: Effects on soil hydraulic conductivity and overland flow production	Ghi_2013_Jik	NA	111.40	27.62	85.55	0	1	0	0	1	0	1	0	0	1	0	0	0	1	0	0	0	0	0	0	0	1	0	0	0	1	0	0	1	0	0	0	0	1	0	0	0	0
40	Gholzom & Gholami	Soil and Water Research	2012	A comparison between natural forests and reforested lands in terms of runoff generation potential and hydrologic response (case study: Kasilian watershed)	Gho_2012_Kas	0	68.00	36.08	53.40	0	0	0	0	1	0	1	0	1	0	0	0	0	0	0	0	0	0	0	0	0	0	1	0	0	1	0	0	0	1	0	0	0	1	0	0	0	0
41	Gonzalez Barney	Third International Symposium on Water Resources, Fifth Caribbean Islands Water Resources Congress, American Water Resources Association	1998	Effects of conifer plantations on water yields and regime in Antioquia Colombia	Gon_1998_Pie	36	22.40	5.76	-74.93	0	0	1	0	0	0	1	0	0	1	0	0	0	0	0	0	0	0	0	0	0	0	0	0	0	0	0	1	0	1	0	0	0	0	0	1	0	0
42	Guo et al.	Journal of Hydrology	2008	Annual and seasonal streamflow responses to climate and land-cover changes in the Poyang Lake basin, China	Guo_2008_Poy	49	15,535.00	28.40	116.25	0	1	1	0	1	0	1	0	0	1	0	0	1	1	1	0	0	0	0	0	0	0	0	0	0	0	0	1	1	1	1	0	0	1	0	0	0	0
43	Guo et al.	Journal of the American Water Resources Association	2014	Decoupling streamflow responses to climate variability and land use/cover changes in a watershed in northern China	Guo_2014_Cha	48	4,855.90	40.60	117.18	1	0	0	1	0	0	1	0	0	1	0	0	0	0	0	0	1	0	0	0	0	0	0	0	0	0	0	1	0	1	0	1	0	1	0	0	0	0
44	Gush et al.	The Southern African Forestry Journal	2002	A new approach to modelling streamflow reductions resulting from commercial afforestation in South Africa	Gus_2002_Cat_II	37	1.90	-29.00	29.25	0	0	1	1	0	0	1	0	0	1	0	0	0	0	0	0	0	0	0	0	0	0	0	0	0	1	0	0	1	1	1	0	1	0	0	0	0	0
					Gus_2002_Wes_D	22	0.40	-23.72	30.07	0	0	1	1	0	0	1	0	0	1	0	0	0	0	0	0	0	0	0	0	0	0	0	0	0	1	0	0	1	1	1	0	1	0	0	0	0	0
					Gus_2002_Lam_A	22	0.31	-33.97	18.95	0	0	1	1	0	0	1	0	0	1	0	0	0	0	0	0	0	0	0	0	0	0	0	0	0	1	0	0	1	1	1	0	1	0	0	0	0	0
					Gus_2002_Lam_B	22	0.66	-33.97	18.95	0	0	1	1	0	0	1	0	1	0	0	0	0	0	0	0	0	0	0	0	0	0	0	0	0	1	0	0	1	1	1	0	1	0	0	0	0	0
					Gus_2002_Nta	5	0.30	-29.05	29.65	0	0	1	1	0	0	1	0	0	1	0	0	0	0	0	0	0	0	0	0	0	0	0	0	0	1	0	0	1	1	1	0	1	0	0	0	0	0
					Gus_2002_Sev	2	0.06	-29.18	30.63	0	0	1	1	0	0	1	0	0	1	0	0	0	0	0	0	0	0	0	0	0	0	0	0	0	1	0	0	1	1	1	0	1	0	0	0	0	0
					Gus_2002_NL	NA	NA	NA	NA	0	0	1	1	0	0	1	0	0	1	0	0	0	0	0	0	0	0	0	0	0	0	0	0	0	1	0	0	0	1	0	0	1	0	0	0	0	0
45	Hanif et al.	Proceedings of the Fiji Symposium	1990	Watershed restoration reduces runoff and sedimentation from comparative watersheds in Pakistan's subtropical scrub zone	Han_1990_Kan_1	13	0.81	33.17	73.37	1	0	0	1	0	0	1	0	0	1	0	0	0	0	0	0	1	0	0	0	0	0	0	0	0	0	0	1	1	0	0	0	0	1	0	0	0	0
46	Hassler et la.	Forest Ecology and Management	2011	Recovery of saturated hydraulic conductivity under secondary succession on former pasture in the humid tropics	Has_2011_Catch	NA	NA	9.21	-79.75	0	1	0	0	0	1	0	0	0	0	0	0	0	0	0	0	0	0	0	0	0	1	0	0	1	0	0	0	1	0	0	0	0	0	0	1	0	0
47	Hayashi et al.	Journal of Environmental Management	2015	Simulation of the reduction of runoff and sediment load resulting from the Gain for Green Program in the Jialingjiang catchment, upper region of the Yangtze River, China	Hay_2015_Wus	NA	NA	30.39	106.22	1	0	0	0	1	0	1	0	0	1	0	0	0	0	0	0	0	0	0	0	0	0	0	0	0	0	0	1	0	1	0	0	0	1	0	0	0	0
					Hay_2015_Bei	NA	NA	30.10	106.35	1	0	0	0	1	0	1	0	0	1	0	0	0	0	0	0	0	0	0	0	0	0	0	0	0	0	0	1	0	1	0	0	0	1	0	0	0	0
					Hay_2015_Meg	NA	NA	30.19	106.44	1	0	0	0	1	0	1	0	0	1	0	0	0	0	0	0	0	0	0	0	0	0	0	0	0	0	0	1	0	1	0	0	0	1	0	0	0	0
					Hay_2015_Xia	NA	NA	30.20	105.90	1	0	0	0	1	0	1	0	0	1	0	0	0	0	0	0	0	0	0	0	0	0	0	0	0	0	0	1	0	1	0	0	0	1	0	0	0	0
48	Herron et al.	Journal of Environmental Management	2003	Modelling the impacts of strategic tree planting on salt loads and flows in the Macquarie River Catchment, NSW, Australia	Her_2003_Mac	20	75,000.00	-32.22	148.25	0	1	0	1	0	0	1	1	1	1	1	0	0	0	0	0	0	0	0	0	0	0	0	0	0	0	0	1	0	1	0	0	0	0	1	0	0	0
49	Herron et al.	Journal of Environmental Management	2002	The effects of large-scale afforestation and climate change on water allocation in the Macquarie River catchment, NSW, Australia	Her_2002_Mac	30	75,000.00	-30.25	146.55	0	1	0	1	0	0	1	1	0	1	0	0	0	0	0	0	0	0	0	0	0	0	0	0	0	0	0	1	0	1	0	0	0	0	1	0	0	0
50	Holmes & Sinclair	Hydrology and Water Resources Symposium, Brisbane, 25-27 November 1986	1986	Water yield from some afforested catchments in Victoria	Hol_1986_REG	14	NA	-38.77	143.48	0	0	1	1	0	0	1	0	0	1	0	0	0	0	0	0	0	0	0	0	0	0	0	0	1	1	1	0	1	0	0	0	0	0	1	0	0	0
51	Hornbeck et al.	Canadian Journal of Forest Research	1997	Summary of water yield experiments at Hubbard Brook Experimental Forest, New Hampshire	Hor_1997_CF	30	0.22	46.93	-71.75	0	1	1	1	0	0	1	0	0	1	0	0	0	0	0	0	0	0	0	0	0	0	0	0	1	0	0	0	1	0	0	1	0	0	0	0	0	1
					Hor_1997_SC	25	0.36	46.93	-71.75	0	1	1	1	0	0	1	0	0	1	0	0	0	0	0	0	0	0	0	0	0	0	0	0	1	0	0	0	1	0	0	1	0	0	0	0	0	1
52	Hornbeck et al.	Journal of Hydrology	1993	Long-term impacts of forest treatments on water yield: a summary for northeastern USA	Hor_1993_Mar	26	0.34	47.52	-93.46	0	1	1	1	0	0	1	0	0	1	0	0	0	0	0	0	0	0	0	0	0	0	0	0	1	0	0	0	1	0	0	0	0	0	0	0	0	1
					Hor_1993_Fen	NA	0.15 - 0.34	39.18	-79.76	0	1	1	1	0	0	1	0	0	1	0	0	0	0	0	0	0	0	0	0	0	0	0	0	1	0	0	0	1	0	0	0	0	0	0	0	0	1
					Hor_1993_Lea	24	0.43 - 0.104	40.66	-77.96	0	1	1	1	0	0	1	0	0	1	0	0	0	0	0	0	1	0	0	0	0	0	0	0	1	0	0	0	1	0	0	0	0	0	0	0	0	1
					Hor_1993_Hub	NA	0.16 -0.36	43.94	-71.71	0	1	1	1	0	0	1	0	0	1	0	0	0	0	0	0	1	0	0	0	0	0	0	0	1	0	0	0	1	0	0	0	0	0	0	0	0	1
53†	Hornbeck	Water Resources Bulletin	1975	Streamflow response to forest cutting an revegetation	Hor_1975_Hub	6	0.16	43.94	-71.71	0	0	1	0	1	0	1	0	0	1	0	0	0	0	0	0	0	0	0	0	0	0	0	0	1	0	0	0	1	0	0	0	0	0	0	0	0	1
54†	Huang & Zhang	Hydrological Processes	2004	Hydrological responses to conservation practices in a catchment of the Loess Plateau, China	Hua_2004_Jia	22	1,117.00	31.87	104.54	0	1	1	0	1	0	1	0	0	1	0	0	0	0	0	0	0	0	0	0	0	0	0	0	0	0	0	1	1	0	0	0	0	1	0	0	0	0
55	Huang et al.	Hydrological Processes	2003	Runoff responses to afforestation in a watershed of the Loess Plateau, China	Hua_2003_Nan_1	24	0.87	37.55	110.28	0	0	1	0	1	0	1	0	0	1	0	0	0	0	0	0	0	0	0	0	0	0	0	0	1	0	0	0	1	0	0	0	0	1	0	0	0	0
56	Hudson et al.	Hydrology and Earth System Sciences	1997	The impact of the growth of new plantation forestry on evaporation and streamflow in the Llanbrynmair catchments	Hud_1997_Lla	12	2.89	52.61	-3.63	0	0	1	0	1	0	1	1	0	0	1	0	0	0	0	0	0	0	0	0	0	0	0	0	0	1	0	0	1	0	0	0	0	0	0	0	1	0
57	Hundecha & Bárdossy	Journal of Hydrology	2004	Modeling of the effect of land use changes on the runoff generation of a river basin through parameter regionalization of a watershed model	Hun_2004_REG	12	400.00 - 2100.00	49.35	7.87	0	0	1	0	0	0	1	0	0	1	0	0	0	0	0	0	0	0	0	0	0	0	0	0	0	0	0	1	1	1	1	0	0	0	0	0	1	0
58	Hurkmans et al.	Water Resources Research	2009	Effects of land use changes on streamflow generation in the Rhine basin	Hur_2009_REG	10	185,000.00	50.50	7.06	0		1	0	1	0	1	1	0	1	0	0	0	0	0	0	0	0	0	0	0	0	0	0	0	0	0	1	0	1	0	0	0	0	0	0	1	0
59	Iroume & Palacios	Journal of Hydrology	2013	Afforestation and changes in forest composition affect runoff in large river basins with pluvial regime and Mediterranean climate, Chile	Iro_2013_Duq	43	1,545.00	-37.54	-72.33	0	0	1	0	0	0	1	0	0	1	0	0	0	1	0	0	0	0	0	0	0	0	0	0	0	1	0	0	1	0	0	0	0	0	0	1	0	0
					Iro_2013_Car	43	94.00	-37.80	-73.38	0	0	1	0	0	0	1	0	0	1	0	0	0	1	0	0	0	0	0	0	0	0	0	0	0	1	0	0	1	0	0	0	0	0	0	1	0	0
					Iro_2013_Mul	43	434.00	-37.72	-72.26	0	0	1	0	0	0	1	0	0	1	0	0	0	1	0	0	0	0	0	0	0	0	0	0	0	1	0	0	1	0	0	0	0	0	0	1	0	0
60	Iroume et al.	Bosque	2010	Runoff and peakflows after clearcutting and the establishment of a new plantation in an experimental catchment, southern Chile	Iro_2010_LaR	12	0.34	40.58	-73.66	0	0	1	0	1	0	1	0	0	1	0	0	0	1	0	0	0	0	0	0	0	0	0	0	0	0	0	1	1	1	1	0	0	0	0	1	0	0
61	Jackson et al.	Science	2005	Trading water for carbon with biological carbon sequestration	Jac_2005_NL	20	0.01 - >10000.00	36.52	-84.34	0	0	1	0	1	0	1	0	0	1	0	0	0	1	0	0	0	0	0	0	0	0	0	0	1	1	1	0	0	1	0	1	0	0	0	0	0	1
62	Jakeman et al.	Journal of Hydrology	1993	An assessment of the dynamic response characteristics of streamflow in the Balquhidder catchments	Jak_1993_Mon	5	NA	56.35	-4.37	0	0	1	0	0	0	1	0	0	0	1	0	0	0	0	0	0	0	0	0	0	0	0	0	0	0	0	1	0	1	0	0	0	0	0	0	1	0
63	Jarabac & Chlebek	Conference paper: Hydrological research basins and the environment.	1990	The results of long-term measurements in four Carpathian Basins	Jar_1990_ZD	60	4.09	49.15	20.42	0	0	0	1	0	0	1	0	0	1	0	0	0	0	0	0	0	0	0	0	0	0	0	0	0	0	0	1	1	0	0	0	0	0	0	0	1	0
					Jar_1990_MR	60	2.07	49.15	20.42	0	0	0	1	1	0	1	0	0	0	1	0	0	0	0	0	0	0	0	0	0	0	0	0	0	0	0	1	1	0	0	0	0	0	0	0	1	0
64	Jones & Post	Water Resouces Research	2004	Seasonal and successional streamglow response to forest cutting and regrowth in the northwest and eastern United States	Jon_2004_Fer	55	0.29 - 0.39	39.18	-79.76	0	1	0	0	0	1	1	0	1	1	0	0	0	0	0	0	0	0	0	0	0	0	0	0	1	0	0	0	0	1	0	0	0	0	0	0	0	1
					Jon_2004_Cow	75	0.12 - 0.61	35.06	-83.43	0	1	0	0	0	1	1	0	1	1	0	0	0	0	0	0	0	0	0	0	0	0	0	0	1	0	0	0	0	1	0	0	0	0	0	0	0	1
					Jon_2004_Hub	45	0.16 - 0.42	43.94	-71.71	0	1	0	0	0	1	1	0	1	1	0	0	0	0	0	0	0	0	0	0	0	0	0	0	1	0	0	0	0	1	0	0	0	0	0	0	0	1
					Jon_2004_And	55	0.09 - 0.96	44.21	-122.26	0	1	0	0	0	1	1	0	1	1	0	0	0	0	0	0	0	0	0	0	0	0	0	0	1	0	0	0	0	1	0	0	0	0	0	0	0	1
					Jon_2004_Coy	20	0.49 - 0.50	43.10	-123.13	0	1	0	0	0	1	1	0	1	0	0	0	0	0	0	0	0	0	0	0	0	0	0	0	1	0	0	0	0	1	0	0	0	0	0	0	0	1
					Jon_2004_Cas	20	0.21 - 0.27	39.36	-123.75	0	1	0	0	0	1	1	0	1	0	0	0	0	0	0	0	0	0	0	0	0	0	0	0	1	0	0	0	0	1	0	0	0	0	0	0	0	1
65	Kalantari et al.	Science of the Total Environment	2014	Quantifying the hydrological impact of simulated changes in land use on peak discharge in a small catchment	Kal_2014_catch_1	1	4.50	59.65	10.94	0	0	0	0	1	0	1	1	0	1	0	0	0	0	0	0	1	0	0	0	0	0	0	0	0	0	0	1	0	1	0	0	0	0	0	0	1	0
67	Kim et al.	Journal of the American Water Resources Association	2014	The influence of forest regrowth on the stream discharge in the North Carolina Piedmont watersheds	Kim_2014_Fla	84	384.00	36.35	-78.93	0	1	0	0	0	1	1	1	0	1	0	0	1	0	0	0	0	0	0	0	0	0	0	0	1	0	0	0	1	1	1	0	0	0	0	0	0	1
68	Kovner	Proceedinigs, Society of American Foresters, Memphis, Tennessee, 1956	1956	Evapotranspiration and water yields following forest cutting and natural regrowth	Kov_1956_Cow	13	0.16	35.06	-83.43	0	1	0	0	0	1	1	1	0	1	0	0	0	0	0	0	0	0	0	1	0	0	0	0	1	0	0	0	1	0	0	0	0	0	0	0	0	1
69	Křeček & Balek	XVII IUFRO World Congress, Japan, 1981	1981	Afforestation and forest treatment effects on streamflow	Kre_1981_Zde	49	NA	49.26	18.14	0	1	0	1	1	0	1	1	0	1	0	0	0	0	0	0	0	0	0	0	0	0	0	0	1	1	1	0	1	1	1	0	0	0	0	0	1	0
					Kre_1981_Kye	49	NA	49.26	18.14	0	0	0	1	1	0	1	1	0	0	1	0	0	0	0	0	0	1	0	0	0	0	0	0	0	1	0	0	1	1	1	0	0	0	0	0	1	0
70	Krestovskii	Water Resources	1984	Effect of felling and reforestation on river discharge in the southern and middle taiga subzones	Kre_1984_Vya	7	NA	59.92	30.35	0	0	0	0	1		1	0	0	1	1	0	0	0	0	0	0	0	0	0	0	0	0	0	1	0	0	0	1	0	0	0	0	0	0	0	1	0

Paper ID	Publication Reference				Study Information			Coordinates		Type of Intervention						Direct Metric		Direct Annual Water Yield Response				Baseflow			Indirect Water Yield Metrics									Forest Type				Data Acquisition Method				Continent					
	Author(s)	Publication name	Publication year	Publication title	Study case name	Years of discharge data	Catchment area (km^2)	Latitude	Longitude	Forest Restoration (explicit)	Forest Restoration (implicit)	Forestry	Afforestation/Forestation	Reforestation	Forest Regrowth	Water yield	Stream flow	Positive	Negative	No change	Unclear	Positive	Negative	Neutral	Increased Flooding/ Peak Flow	Decreased Flooding/ Peak Flow	Neutral Flooding/ Peak Flow	Increased GW level/ recharge	Decreased GW level/ recharge	Neutral GW level/ recharge	Increased soil infiltration / capacity	Decreased soil infiltration / capacity	Neutral soil infiltration / capacity	Native Forest	Non-native Forest	Mixed	ND Species	Field-Data	Model	Mixed	Historical Data/ Meta-data	Africa	Asia+ Middle East	Oceania	South+ Central America	Europe	North America
71	Krishnaswamy et al.	Journal of Hydrology	2012	The rain-runoff response of tropical humid forest ecosystems to use and reforestation in the western ghats of India	Kri_2012_Aca	2	0.07 - 0.23	15.09	74.39	0	0	1	0	0	1	1	1	0	0	0	1	0	0	0	0	0	0	0	0	0	1	0	0	0	1	0	0	1	0	0	1	0	1	0	0	0	0
72	Krishnaswamy et al.	Journal of Hydrology	2013	The groundwater recharge response and hydrologic services of tropical humid forest ecosystems to use and reforestation: Support for the "infiltration-evapotranspiration trade-off hypothesis"	Kri_2013_Aca	2	0.07 - 0.23	15.09	74.39	0	1	0	0	1	0	1	0	0	1	0	0	0	0	0	0	1	0	1	0	0	1	0	0	0	1	0	0	1	1	1	0	0	1	0	0	0	0
73	Kuczera	Journal of Hydrology	1987	Prediction of water yield reductions following a bushfire in ash-mixed species eucalypt forest	Kuc 1987 Wat	150	100.94	-37.67	145.49	0	1	0	0	0	1	1	0	1	1	0	0	0	0	0	0	0	0	0	0	0	0	0	0	1	0	0	0	1	1	1	0	0	0	1	0	0	0
					Kuc_1987_Osh	150	127.43	-37.71	145.76	0	1	0	0	0	1	1	0	0	1	0	0	0	0	0	0	0	0	0	0	0	0	0	0	1	0	0	0	1	1	1	0	0	0	1	0	0	0
					Kuc_1987_Gra	150	26.08	-37.81	144.96	0	1	0	0	0	1	1	0	1	1	0	0	0	0	0	0	0	0	0	0	0	0	0	0	1	0	0	0	1	1	1	0	0	0	1	0	0	0
					Kuc_1987_Don	150	14.10	-37.81	144.96	0	1	0	0	0	1	1	0	1	1	0	0	0	0	0	0	0	0	0	0	0	0	0	0	1	0	0	0	1	1	1	0	0	0	1	0	0	0
					Kuc_1987_Mur	150	NA	-37.81	144.96	0	1	0	0	0	1	1	0	1	1	0	0	0	0	0	0	0	0	0	0	0	0	0	0	1	0	0	0	1	1	1	0	0	0	1	0	0	0
					Kuc_1987_Saw	150	4.16	-37.81	144.96	0	1	0	0	0	1	1	0	1	1	0	0	0	0	0	0	0	0	0	0	0	0	0	0	1	0	0	0	1	1	1	0	0	0	1	0	0	0
					Kuc_1987_Cor	150	19.32	-37.81	144.96	0	1	0	0	0	1	1	0	0	0	0	1	0	0	0	0	0	0	0	0	0	0	0	0	1	0	0	0	1	1	1	0	0	0	1	0	0	0
					Kuc_1987_Tho	150	907.00	-37.81	144.96	0	1	0	0	0	1	1	0	0	0	1	0	0	0	0	0	0	0	0	0	0	0	0	0	1	0	0	0	1	1	1	0	0	0	1	0	0	0
74	Lane et al.	Journal of Hydrology	2005	The response of flow duration curves to afforestation	Lan_2005_Tra	NA	87.00	-38.20	146.53	0	0	1	1	0	0	1	0	0	1	0	0	0	1	0	0	0	0	0	0	0	0	0	0	0	1	0	0	1	1	1	0	0	0	1	0	0	0
					Lan_2005_Cat2	11	1.90	-28.93	29.13	0	0	1	1	0	0	1	0	0	1	0	0	0	1	0	0	0	0	0	0	0	0	0	0	0	1	0	0	1	1	1	0	1	0	0	0	0	0
					Lan_2005_Cat3	11	1.39	-28.93	29.13	0	0	1	1	0	0	1	0	0	1	0	0	0	1	0	0	0	0	0	0	0	0	0	0	0	1	0	0	1	1	1	0	1	0	0	0	0	0
					Lan_2005_Red	12	1.95	-31.83	147.90	0	0	1	1	0	0	1	0	0	1	0	0	0	1	0	0	0	0	0	0	0	0	0	0	0	1	0	0	1	1	1	0	0	0	1	0	0	0
					Lan_2005_Pin	20	3.20	-36.81	145.08	0	0	1	1	0	0	1	0	0	1	0	0	0	1	0	0	0	0	0	0	0	0	0	0	0	1	0	0	1	1	1	0	0	0	1	0	0	0
					Lan_2005_Ste	NA	0.18	-37.03	145.40	0	0	1	1	0	0	1	0	0	1	0	0	0	1	0	0	0	0	0	0	0	0	0	0	0	1	0	0	1	1	1	0	0	0	1	0	0	0
					Lan_2005_Gle	NA	3.10	-45.88	169.76	0	0	1	1	0	0	1	0	0	1	0	0	0	1	0	0	0	0	0	0	0	0	0	0	0	1	0	0	1	1	1	0	0	0	1	0	0	0
					Lan_2005_LamA	NA	0.31	-33.97	18.93	0	0	1	1	0	0	1	0	0	1	0	0	0	1	0	0	0	0	0	0	0	0	0	0	0	1	0	0	1	1	1	0	1	0	0	0	0	0
					Lan_2005_LamB	NA	0.66	-33.97	18.93	0	0	1	1	0	0	1	0	0	1	0	0	0	1	0	0	0	0	0	0	0	0	0	0	0	1	0	0	1	1	1	0	1	0	0	0	0	0
					Lan_2005_Bie	20	0.27	-33.98	18.95	0	0	1	1	0	0	1	0	0	1	0	0	0	1	0	0	0	0	0	0	0	0	0	0	0	1	0	0	1	1	1	0	1	0	0	0	0	0
75	Lane & Mackay	Forest Ecology and Management	2001	Streamflow response of mixed-species eucalypt forests to patch cutting and thinning treatments	Lan_2001_Wic	10	0.68	-34.70	149.50	0	0	1	0	0	1	1	1	0	1	0	0	0	0	0	0	0	0	0	0	0	0	0	0	1	0	0	0	1	0	0	0	0	0	1	0	0	0
					Lan_2001_Wil	10	0.86	-34.70	149.50	0	0	1	0	0	1	1	1	0	1	0	0	0	0	0	0	0	0	0	0	0	0	0	0	1	0	0	0	1	0	0	0	0	0	1	0	0	0
76	Lewis et al.	Hydrological Processes	2013	How does afforestation affect the hydrology of a blanket peatland? A modelling study	Lew_2013_catch_1	1	0.76	51.97	-9.90	0	0	0	1	0	0	1	1	0	1	0	0	0	0	0	0	0	0	0	1	0	0	0	0	0	1	0	0	1	1	1	0	0	0	0	0	1	0
77	Li et al.	Journal of Hydrology	2012	Separating effects of vegetation change and climate variability using hydrological modelling and sensitivity-based approaches	Li_2012_Cra	35	698.00	-38.21	141.68	0	0	0	1	0	0	1	1	0	1	0	0	0	0	0	0	0	0	0	0	0	0	0	0	0	1	0	0	1	1	1	0	0	0	1	0	0	0
					Li_2012_Dar	23	760.00	-38.21	141.68	0	0	0	1	0	0	1	1	0	1	0	0	0	0	0	0	0	0	0	0	0	0	0	0	0	1	0	0	1	1	1	0	0	0	1	0	0	0
					Li_2012_Tin	47	1,174.00	-25.61	152.76	0	0	0	1	0	0	1	1	0	1	0	0	0	0	0	0	0	0	0	0	0	0	0	0	0	1	0	0	1	1	1	0	0	0	1	0	0	0
78†	Li et al.	Hydrological Processes	2014	Long-term hydrological response to reforestation in a large watershed in southeastern China	Li_2014_Lia	38	579.00	26.43	115.68	0	1	0	0	1	0	1	1	0	1	0	0	0	0	0	0	0	0	0	0	0	0	0	0	0	0	0	1	1	0	0	0	0	1	0	0	0	0
79†	Lima et al.	Forest Service Research Paper NE-392	1978	Natural reforestation reclaims a watershed: A case history from West Virgina	Lim_1978_catch_9	13	0.12	39.11	-79.67	0	1	0	0	1	0	1	1	0	0	1	0	0	0	0	0	0	0	0	0	0	0	0	0	1	0	0	0	0	0	0	0	0	0	0	0	0	1
					Lim_1978_catch_8	13	0.19	39.11	-79.67	0	1	0	0	1	0	1	1	0	0	1	0	0	0	0	0	0	0	0	0	0	0	0	0	1	0	0	0	0	0	0	0	0	0	0	0	0	1
80	Little et al.	Journal of Hydrology	2009	Revealing the impact of forest exotic plantations on water yield in large scale watersheds in South-Central Chile	Lit_2009_CQA	43	707.70	-35.57	-72.21	0	0	1	0	0	0	1	1	0	1	0	0	0	1	0	0	0	0	0	0	0	0	0	0	0	1	0	0	1	0	0	0	0	0	0	1	0	0
					Lit_2009_PPN	43	252.50	-35.95	-72.45	0	0	1	0	0	0	1	1	0	1	0	0	0	1	0	0	0	0	0	0	0	0	0	0	0	1	0	0	1	0	0	0	0	0	0	1	0	0
81	Liu et al.	Journal of the American Water Resources Association	2008	Effects of land-use and land-cover change on evapotranspiration and water yield in China during 1900-2000	Liu_2008_NL	10	NA	35.51	104.25	0	1	0	0	1	0	1	0	1	0	0	0	0	0	0	0	0	0	0	0	0	0	0	0	0	0	0	1	0	1	0	0	0	1	0	0	0	0
82†	Liu et al.	Ecohydrology	2015	How do climate and forest changes affect long-term streamflow dynamics? A case study in the upper reach of Poyang River basin	Liu_2015_Mei	49	6,983.20	26.52	115.93	0	1	0	0	1	0	1	1	0	1	0	0	0	0	0	0	0	0	0	0	0	0	0	0	1	0	0	0	1	0	0	0	0	1	0	0	0	0
83	Liu et al.	Journal of Hydrologic Engineering	2013	Impacts of land-use and climate changes on hydrologic processes in the Qingyi River Watershed, China	Liu_2013_Qin	25	13,262.93	30.01	102.86	0	1	0	1	0	0	1	0	0	0	1	0	0	1	0	0	0	0	0	0	0	0	0	0	0	0	0	1	0	1	0	0	0	1	0	0	0	0
84	Liu et al.	Journal of the American Water Resources Association	2008	Modeling the potential of the northern China forest shelterbelt in improving hydroclimate conditions	Liu_2008_REG	1	9,000,000.00	39.86	105.00	0	1	0	1	0	0	1	0	1	1	0	0	0	0	0	0	0	0	0	0	0	0	0	0	0	0	0	1	0	1	0	0	0	1	0	0	0	0
85	Liu et al.	Land Degradation and Development	2014	Land use and climate changes and their impacts on runoff in the Yarlung Zangbo river basin, China	Liu_2014_whole	26	NA	29.13	87.63	0	1	0	1	0	0	1	1	1	0	0	0	0	0	0	0	0	0	0	0	0	0	0	0	0	0	0	1	0	0	0	0	0	1	0	0	0	0
86	Liu et al.	Land Degradation and Development	2014	Land use and climate changes and their impacts on runoff in the Yarlung Zangbo river basin, China	Liu_2014_reg_2	26	NA	29.34	89.97	0	1	0	1	0	0	1	1	1	0	0	0	0	0	0	0	0	0	0	0	0	0	0	0	0	0	0	1	0	0	0	0	0	1	0	0	0	0
					Liu_2014_reg_3	26	NA	29.23	92.01	0	1	0	1	0	0	1	1	1	0	0	0	0	0	0	0	0	0	0	0	0	0	0	0	0	0	0	1	0	0	0	0	0	1	0	0	0	0
					Liu_2014_reg_4	26	NA	29.47	94.75	0	1	0	1	0	0	1	1	1	0	0	0	0	0	0	0	0	0	0	0	0	0	0	0	0	0	0	1	0	0	0	0	0	1	0	0	0	0
87	Locatelli & Vignola	Forest Ecology and Management	2009	Managing watershed services of tropical forests and plantations: Can meta-analyses help?	Loc_2009_REV	41	<1.00 - >1.00	25.12	99.34	0	0	1	0	0	0	1	0	0	1	0	0	0	1	0	0	0	0	0	0	0	0	0	0	0	1	0	0	1	0	0	0	1	1	0	1	0	0
88	López-Moreno et al.	Hydrological Sciences Journal	2006	Trends in high flows in the central Spanish Pyrenees: response to climatic factors or to land-use change?	Lop_2006_REG	40	NA	42.34	1.43	0	1	0	0	1	0	1	0	0	1	0	0	1	0	1	0	1	0	0	0	0	0	0	0	1	0	0	0	1	0	0	0	0	0	0	0	1	0
89	Lorz et al.	Forest Ecology and Management	2007	Considering spatial distribution and functionality of forests in a modeling framework for river basin management	Lor_2007_REG	NA	NA	49.99	10.35	0	0	0	0	1	0	1	0	0	1	0	0	0	0	0	0	0	0	0	0	0	0	0	0	0	0	0	1	1	0	0	0	0	0	0	0	1	0
90	Ma et al.	Hydrological Processes	2009	Response of hydrological processes to land-cover and climate changes in Kejie watershed, south-west China	Ma_2009_Kej	40	1,755.00	25.12	99.34	0	1	0	0	1	0	1	1	0	1	0	0	1	1	0	0	0	0	0	0	0	0	0	0	0	1	0	0	0	1	0	0	0	1	0	0	0	0
91	Ma et al.	Hydrological Processes	2010	Sensitivity of streamflow from a Himalayan catchment to plausible changes in land cover and climate	Ma_2010_Kej	138	1,755.00	25.12	99.34	0	0	0	1	0	0	1	0	0	1	0	0	0	0	0	0	0	0	0	0	0	0	0	0	0	0	0	1	0	1	0	0	0	1	0	0	0	0
92	Magilligan & Stamp	Annals of the Association of American Geographers	2010	Historical land-cover changes and hydrogeomorphic adjustment in a small Georgia watershed	Mag_1020_Tur	31	60.00	32.11	-84.82	0	1	0	0	0	1	1	0	0	0	0	1	0	0	0	0	0	0	0	0	0	0	0	0	1	0	0	0	0	1	0	0	0	0	0	0	0	1
93	Mathur et al.	The Indian Forester	1976	Effect of clearfelling and reforestation on runoff and peak rates in small watersheds	Mat_1976_W1F	13	0.01	30.35	77.83	0	0	1	0	1	0	1	0	0	1	0	0	0	0	0	0	1	0	0	0	0	0	0	0	0	1	0	0	1	0	0	0	0	1	0	0	0	0
94	McGuinness & Harrold	Water Resources Research	1971	Reforestation influences on small watershed streamflow	Mcg_1971_Nor	25	0.18	40.36	-81.79	0	1	0	0	1	0	1	1	0	1	0	0	0	1	0	0	0	0	0	0	0	0	0	0	0	1	0	0	1	0	0	0	0	0	0	0	0	1
95	McVicar et al.	Forest Ecology and Management	2007	Developing a decision support tool for China's re-vegetation program: Simulating regional impacts of afforestation on average annual streamflow in the Loess Plateau	Mcv_2007_REG	NA	NA	36.96	107.54	0	1	0	1	0	0	1	1	0	1	0	0	0	0	0	0	0	0	0	0	0	0	0	0	0	0	0	1	0	1	0	0	0	1	0	0	0	0
96	Meuser	Agricultural and Forest Meteorology	1990	Effects of afforestation on runoff characteristics	Meu_1990_Kro	4	0.33	50.62	8.64	0	0	0	1	0	0	1	0	0	1	0	0	0	0	0	0	0	0	0	1	0	0	0	0	1	0	0	0	0	1	0	0	0	0	0	0	1	0
97	Montenegro & Ragab	Journal of Hydrology	2012	Impact of possible climate and land use changes in the semi arid regions: A case study from North Eastern Brazil	Mon_2012_Tap	89	470.50	-8.17	-35.53	0	1	0	0	1	0	1	0	0	1	0	0	0	0	0	0	0	0	0	1	0	0	1	0	0	0	0	1	0	1	0	0	0	0	0	1	0	0
98	Muñoz-Villers et al.	Journal of Hydrology	2012	Water balances of old-growth and regenerating montane cloud forests in central Veracruz, Mexico	Mun_2012_SEC	2	0.12	19.49	-96.78	0	1	0	0	0	1	1	1	1	0	0	0	0	0	0	0	0	1	0	0	0	0	0	0	1	0	0	0	1	0	0	0	0	0	0	1	0	0
99	Muñoz-Villers & McDonnell	Hydrology and Earth System Sciences	2013	Land use change effects on runoff generation in a humid tropical montane cloud forest region	Mun_2013_SEC	2	0.12	19.49	-96.78	0	0	0	0	0	1	1	1	0	1	0	0	0	1	0	0	0	0	0	0	0	1	0	0	1	0	0	0	1	0	0	0	0	0	0	1	0	0
100†	Mwendera	Forest Ecology and Management	1994	Effect on the water yield of the Luchelemu catchment in Malawi of replacing indigenous grasses with timber plantations	Mwe_1994_Luc	17	13.30	-11.69	34.02	0	0	1	0	0	0	1	0	0	1	0	0	0	1	0	0	0	1	0	0	0	0	0	0	0	1	0	0	1	0	0	0	1	0	0	0	0	0
101	Nänni	South African Forestry Journal	1970	The effect of afforestation on streamflow at Cathedral Peak: Report No. 1	Nan_1970_II	20	1.90	-28.93	29.13	0	0	0	1	0	0	1	1	0	1	0	0	0	0	0	0	0	0	0	0	0	0	0	0	0	1	0	0	1	0	0	0	1	0	0	0	0	0
					Nan_1970_III	20	1.42	-28.93	29.13	0	0	0	1	0	0	1	1	0	1	0	0	0	0	0	0	0	0	0	0	0	0	0	0	0	1	0	0	1	0	0	0	1	0	0	0	0	0
102	Nosetto et al.	Agriculture, Ecosystems and Environment	2012	The hydrologic consequences of land cover change in central Argentina	Nos_2012_UND	NA	NA	-31.49	-60.38	0	0	1	0	0	0	0	0	0	0	0	0	0	0	0	0	0	0	0	0	0	0	0	0	0	1	0	0	0	1	0	0	0	0	0	1	0	0
103	Otero et al.	Land Use Policy	2011	Loss of water availability and stream biodiversity under land abandonment in a Mediterranean catchment (Olzinelles, NE Spain)	Ote_2011_Olz	30	8.28	41.68	2.52	0	1	0	0	0	1	1	1	0	0	1	0	0	0	0	0	0	0	0	0	0	0	0	0	1	1	1	0	0	1	0	0	0	0	0	0	1	0
104	Ouyang et al.	Ecological Engineering	2015	Estimating effects of reforestation on nitrogen and phosphorus load reductions in the Lower Yazoo River Watershed, Mississippi	Ouy_2015_LYRW	10	618.00	32.33	-90.90	0	1	0	0	1	0	1	1	0	1	0	0	0	0	0	0	0	0	0	0	0	0	0	0	0	0	0	1	0	1	0	0	0	0	0	0	0	1
105	Pearce et al.	Forest Hydrology and Watershed Management Proceedings of the Vancouver Symposium	1987	Reforestation: on-site effects on hydrology and erosion, eastern Raukumara Range, New Zealand	Pea_1987_Wai	NA	NA	-38.38	177.83	0	1	0	0	1	0	1	0	0	1	0	0	0	0	0	0	0	0	0	0	0	0	0	0	0	1	0	0	1	0	0	0	0	0	1	0	0	0
106	Perry et al.	Forest Ecology and Management	2001	Impacts of short-rotation hybrid poplar plantations on regional water yield	Per_2001_Red	1	NA	47.58	-96.10	0	0	0	0	1	0	1	1	0	0	1	0	0	1	0	0	0	0	0	0	0	0	0	0	0	1	0	0	0	1	0	0	0	0	0	0	0	1
107†	Pitman	Journal of Hydrology	1978	Trends in streamflow due to upstream land-use changes	Pit_1978_Que	23	200.00	-25.75	28.23	0	0	0	1	0	0	1	1	0	1	0	0	0	0	0	0	0	0	0	0	0	1	0	0	0	1	0	1	0	1	0	0	1	0	0	0	0	0
					Pit_1978_Kla	33	NA	NA	NA	0	0	0	1	0	0	1	1	0	1	0	0	0	0	0	0	0	0	0	0	0	0	0	0	0	1	0	1	1	0	0	0	1	0	0	0	0	0
108	Qiu et al.	Journal of Environmental Quality	2011	Effects of the "Conversion of Cropland to Forest and Grassland Program" on the water budget of the Jinghe River Catchment in China	Qiu_2011_26	4	1,299.84	35.35	108.70	1	0	0	0	1	0	1	1	1	0	0	0	0	0	0	0	0	0	0	0	0	1	0	0	0	0	0	1	0	1	0	0	0	1	0	0	0	0
					Qiu_2011_15	4	473.03	35.75	107.49	1	0	0	0	1	0	1	1	1	0	0	0	0	0	0	0	0	0	0	0	0	1	0	0	0	0	0	1	0	1	0	0	0	1	0	0	0	0
					Qiu_2011_4	4	917.37	36.70	107.05	1	0	0	0	1	0	1	1	1	0	0	0	0	0	0	0	0	0	0	0	0	1	0	0	0	0	0	1	0	1	0	0	0	1	0	0	0	0
109	Querner et al.	Concepts and Models for Sustainable Water Resource Management, *in* FRIEND'97 Regional Hydrology (IAHS Publication no. 246)	1997	Impact of land-use, climate change and groundwater abstraction on streamflow droughts using physically-based models	Que_1997_Hup	23	6.50	52.09	6.62	0	0	0	1	0	0	1	0	0	1	0	0	0	0	0	0	0	0	0	0	0	0	0	0	0	0	0	1	0	1	0	0	0	0	0	0	1	0
					Que_1997_Mon	3	7.70	56.34	-4.48	0	0	0	1	0	0	1	0	0	1	0	0	0	0	0	0	0	0	0	0	0	0	0	0	0	0	0	1	0	1	0	0	0	0	0	0	1	0
110	Ray et al.	Environmental Management	2010	The impact of future land use scenarios on runoff volumes in the Muskegon River Watershed	Ray_2010_Mus	40	7,057.00	43.27	-88.23	0	0	0	0	1	1	1	0	0	1	0	0	0	0	0	0	0	0	0	0	0	0	0	0	1	0	0	0	0	1	0	0	0	0	0	0	0	1
111	Rennie	Nature	1957	Effect of afforestation of catchment areas upon water yield	Ren_1957_Cle	31	NA	54.52	-1.02	0	0	0	1	0	0	1	1	0	1	0	0	0	0	0	0	0	0	0	0	0	0	0	0	0	1	0	0	1	0	0	0	0	0	0	0	1	0
112	Ristic & Macan	Proceedings of Rabat Symposium S6, IAHS Pibl. No 245.	1997	The impact of erosion control measures on runoff processes	Ris_1997_MI	15	0.08	43.56	20.80	0	1	0	0	0	0	1	0	0	0	0	1	0	0	0	0	0	0	0	0	0	0	0	0	0	1	0	0	1	0	0	0	0	0	0	0	1	0
					Ris_1997_MIII	15	0.08	43.56	20.80	0	1	0	0	0	0	1	0	0	1	0	0	0	1	0	0	0	0	0	0	0	0	0	0	0	1	0	0	1	0	0	0	0	0	0	0	1	0
113	Robinson et al.	Hydrological Sciences Journal	1991	A comparison of the hydrology of moorland under natural conditions, agricultural use and forestry	Rob_1991_FM/S	18	0.03	47.80	12.43	0	1	1	1	0	0	1	0	0	1	0	0	0	1	0	0	1	0	0	1	0	0	0	0	0	1	0	0	1	0	0	0	0	0	0	0	1	0
					Rob_1991_FM/N	18	0.03	47.80	12.43	0	1	1	1	0	0	1	0	0	1	0	0	0	1	0	0	1	0	0	1	0	0	0	0	0	1	0	0	1	0	0	0	0	0	0	0	1	0
114	Rodríguez-Suárez et al.	Hydrological Processes	2014	Assessing the influence of afforestation with Eucalyptus globulus on hydrological response from a small catchment in northwestern Spain using the HBV hydrological model	Rod_2014_Mab	9	0.42	43.15	-8.35	0	0	1	1	0	0	1	1	0	1	0	0	0	0	0	0	0	0	0	0	0	0	0	0	0	1	0	0	0	1	0	0	0	0	0	0	1	0
115	Rodríguez-Suárez et al.	Journal of Hydrology	2011	Influence of Eucalyptus globulus plantation growth on water table levels and low flows in a small catchment	Rod_2011_Mab	8	0.11	43.15	-8.34	0	0	1	1	0	0	1	1	0	1	0	0	0	0	0	0	0	0	0	1	0	0	0	0	0	1	0	0	1	0	0	0	0	0	0	0	1	0
116	Rothacher	The Journal of Forestry	1953	White Hollow Watershed management: 15 years of progress in character of forest, runoff, and streamflow	Rot_1953_Whi	14	6.94	36.38	-83.88	0	1	0	0	1	0	1	0	0	0	1	0	0	0	0	0	1	0	0	0	0	1	0	0	1	1	1	0	1	0	0	0	0	0	0	0	0	1
117	Ruprecht & Stoneman	Journal of Hydrology	1993	Water yield issues in the jarrah forest of south-western Australia	Rup_1993_Wel_1	14	NA	-33.20	116.02	0	1	0	0	1	1	1	1	0	1	0	0	0	0	0	0	0	0	0	0	0	0	0	0	1	0	0	0	1	0	0	0	0	0	1	0	0	0
118	Sahin & Hall	Journal of Hydrology	1996	The effects of afforestation and deforestation on water yields	Sah_1996_SYN	NA	NA	NA	NA	0	0	0	1	0	0	1	1	0	1	0	0	0	0	0	0	0	0	0	0	0	0	0	0	0	0	0	1	1	0	0	1	0	0	0	0	0	0
119	Santillian et al.	International Archives of Photogrammetry, Remote Sensing and Spatial Information Sciences	2010	Detection of 25-year land-cover change in a critical watershed in southern Philippines using landsat Mss and Etm + images: Importance in watershed rehabilitation	San 2010 Tag 1	25	NA	9.06	125.72	1	0	1	0	1	0	1	1	0	0	1	0	0	0	0	0	0	0	0	0	0	0	0	0	1	1	1	0	0	1	0	0	0	1	0	0	0	0
					San 2010 Tag 2	25	NA	9.06	125.72	1	0	1	0	1	0	1	1	0	0	1	0	0	0	0	0	0	0	0	0	0	0	0	0	1	1	1	0	0	1	0	0	0	1	0	0	0	0
					San 2010 Tag 3	25	NA	9.05	125.70	1	0	1	0	1	0	1	1	0	1	0	0	0	0	0	0	0	0	0	0	0	0	0	0	1	1	1	0	0	1	0	0	0	1	0	0	0	0
					San 2010 Tag 4	25	NA	9.05	125.70	1	0	1	0	1	0	1	1	0	1	0	0	0	0	0	0	0	0	0	0	0	0	0	0	1	1	1	0	0	1	0	0	0	1	0	0	0	0
					San 2010 Tag 5	25	8.75	9.03	125.68	1	0	1	0	1	0	1	1	0	1	0	0	0	0	0	0	0	0	0	0	0	0	0	0	1	1	1	0	0	1	0	0	0	1	0	0	0	0
					San 2010 Tag 6	25	16.48	9.01	125.66	1	0	1	0	1	0	1	1	0	1	0	0	0	0	0	0	0	0	0	0	0	0	0	0	1	1	1	0	0	1	0	0	0	1	0	0	0	0
					San 2010 Tag 7	25	3.22	9.01	125.66	1	0	1	0	1	0	1	1	0	1	0	0	0	0	0	0	0	0	0	0	0	0	0	0	1	1	1	0	0	1	0	0	0	1	0	0	0	0
					San 2010 Tag 8	25	NA	9.01	125.66	1	0	1	0	1	0	1	1	0	1	0	0	0	0	0	0	0	0	0	0	0	0	0	0	1	1	1	0	0	1	0	0	0	1	0	0	0	0
					San 2010 Tag 9	25	3.46	9.01	125.65	1	0	1	0	1	0	1	1	0	1	0	0	0	0	0	0	0	0	0	0	0	0	0	0	1	1	1	0	0	1	0	0	0	1	0	0	0	0
					San 2010 Tag 10	25	9.06	8.98	125.63	1	0	1	0	1	0	1	1	0	1	0	0	0	0	0	0	0	0	0	0	0	0	0	0	1	1	1	0	0	1	0	0	0	1	0	0	0	0
					San 2010 Tag 11	25	16.54	8.98	125.63	1	0	1	0	1	0	1	1	0	1	0	0	0	0	0	0	0	0	0	0	0	0	0	0	1	1	1	0	0	1	0	0	0	1	0	0	0	0
120	Savill & Weatherup	Forestry	1973	The effect of afforestation on water runoff in the Woodburn Catchment area	Sav_1973_Woo_1	11	1.12	53.92	-5.88	0	0	0	0	0	0	1	0	0	0	1	0	0	0	0	0	0	0	0	0	0	0	0	0	0	1	0	0	1	0	0	0	0	0	0	0	1	0
121	Schneider & Ayer	US Geological Suvey Water-Supply Paper # 1602	1961	Effect of reforestation on streamflow in central New York	Sch_1961_Sha	23	8.08	42.75	-76.03	0	1	0	0	1	1	1	1	0	1	0	0	0	0	0	0	1	0	0	0	0	0	0	0	0	1	0	0	1	0	0	0	0	0	0	0	0	1
122	Schulze & George	Forest Hydrology and Watershed Management Proceedings of the Vancouver Symposium.	1987	Simulation of effects of forest growth water yield with a dynamic process-based user model	Sch_1987_CII	18	1.95	-29.00	29.25	0	0	1	1	0	0	1	1	0	1	0	0	0	0	0	0	0	0	0	0	0	0	0	0	0	1	0	0	0	1	0	0	1	0	0	0	0	0
123	Scott	Canadian Journal of Forest Research	1999	Managing riparian zone vegetation to sustain streamflow: results of paired catchment experiements in South Africa	Sco_1999_Wit2	4	1.36	-23.07	30.07	0	0	1	1	0	0	1	0	0	1	0	0	0	0	0	0	0	0	0	0	0	0	0	0	0	1	0	0	1	0	0	0	1	0	0	0	0	0
					Sco_1999_WesD	4	0.40	-23.07	30.07	0	0	1	1	0	0	1	0	1	0	0	0	0	0	0	0	0	0	0	0	0	0	0	0	0	1	0	0	1	0	0	0	1	0	0	0	0	0
					Sco_1999_Bie	4	0.27	-23.07	30.07	0	0	0	0	0	1	1	0	1	0	0	0	0	0	0	0	0	0	0	0	0	0	0	0	0	1	0	0	1	0	0	0	1	0	0	0	0	0
124	Scott et al.	Water SA	1998	Forestry and streamflow reductions in South Africa: A reference system for assessing extent and distribution	Sco_1998_REG	NA	5,308 - 378,241	-30.97	23.25	0	0	1	0	0	0	1	0	0	1	0	0	1	0	1	0	0	0	0	0	0	0	0	0	0	1	0	0	0	1	0	0	1	0	0	0	0	0
125	Scott & Lesch	Journal of Hydrology	1997	Streamflow responses to afforestation with Eucalyptus grandis and Pinus patula and to felling in the Mokobulaan experimental catchmanets, South Africa	Sco_1997_Mok_A	30	0.26	-25.28	30.57	0	0	1	1	0	0	1	0	0	1	0	0	0	0	0	0	0	0	0	0	0	0	0	0	0	1	0	0	1	0	0	0	1	0	0	0	0	0
					Sco_1997_Mok_B	30	0.35	-25.28	30.57	0	0	1	1	0	0	1	0	0	1	0	0	0	0	0	0	0	0	0	0	0	0	0	0	0	1	0	0	1	0	0	0	1	0	0	0	0	0
126	Scott & Prinsloo	Water Resources Research	2008	Longer-term effects of pine and eucalypt plantations on streamflow	Sco_2008_Wes	30	0.33 - 0.40	-23.73	30.07	0	0	1	1	0	0	1	0	0	1	1	0	0	0	0	0	1	0	0	0	0	0	0	0	0	1	0	0	1	0	0	0	1	0	0	0	0	0
127	Singh & Mishra	Journal of Hydrology	2012	Spatiotemporal analysis of the effects of forest covers on water yield in the western Ghats of peninsular India	Sin_2012_Vai_U	38	160.80	19.15	72.89	0	1	0	0	1	0	1	0	0	1	0	0	0	0	0	0	0	0	0	0	0	0	0	0	0	1	0	0	1	0	0	0	0	1	0	0	0	0
128	Smith	Journal of Environmental Quality	1992	Riparian afforestation effects on water yields and water quality in pasture catchments	Smi_1992_C4	17	0.03	-41.37	173.07	0	1	0	1	0	0	1	0	0	1	0	0	0	0	0	1	1	1	0	0	0	0	0	0	0	1	0	0	1	0	0	0	0	0	1	0	0	0
129	Smith & Scott	Water SA	1992	The effects of afforestation on low flows in various regions of South-Africa	Smi 1992 Mok A-B	20	0.26 - 0.40	-25.28	30.07	0	0	1	1	0	0	1	0	0	1	0	0	0	0	0	0	1	0	0	0	0	0	0	0	0	1	0	0	0	1	0	0	1	0	0	0	0	0
					Smi_1992_Cat_III	23	0.99 - 1.42	-29.00	29.25	0	0	1	1	0	0	1	0	0	1	0	0	0	0	0	0	1	0	0	0	0	0	0	0	0	1	0	0	0	1	0	0	1	0	0	0	0	0
					Smi_1992_Jon_B	23	0.66 - 2.01	-33.95	18.25	0	0	1	1	0	0	1	0	0	1	0	0	0	0	0	0	1	0	0	0	0	0	0	0	0	1	0	0	0	1	0	0	1	0	0	0	0	0
130	Sun et al.	Journal of Hydrology	2006	Potential water yield reduction due to forestation across China	Sun_2006_NL	70	NA	35.85	104.18	1	0	0	1	0	0	1	0	0	1	0	0	0	0	0	0	0	0	0	0	0	0	0	0	0	0	0	1	0	1	0	0	0	1	0	0	0	0
131	Swank et al.	Forest Ecology and Management	2001	Long-term hydrologic and water quality responses following commercial clearcutting of mixed hardwoods on southern Appalachian catchment	Swa_2001_WS7	28	0.59	35.05	-83.42	0	1	0	0	0	1	1	0	1	1	0	0	1	0	1	0	1	0	0	0	0	0	0	0	1	0	0	0	0	1	0	0	0	0	0	0	0	1
132	Swank & Helvey	Int. Assoc. Hydrol. Sci.	1970	Reduction of streamflow increases following regrowth of clearcut hardwood forests	Swa_1970_37	35	0.44	35.05	-83.42	0	1	0	0	0	1	1	0	0	0	0	1	0	0	0	0	0	0	0	0	0	0	0	0	1	0	0	0	1	0	0	0	0	0	0	0	0	1
					Swa_1970_13	35	0.16	35.05	-83.42	0	1	0	0	0	1	1	0	0	1	0	0	0	0	0	0	0	0	0	0	0	0	0	0	1	0	0	0	1	0	0	0	0	0	0	0	0	1
133	Thongmee & Vannaprasert	Proceedings of the Fiji Symposium	1990	Soil and water losses on plots with different land use in the Phu Wlang watershed	Tho_1990_Phu	0	NA	16.67	102.49	0	0	1	0	0	0	1	0	0	1	0	0	0	0	0	0	0	0	0	0	0	0	0	0	0	1	0	0	1	0	0	0	0	1	0	0	0	0
134	Townsend et al.	Forest Policy and Economics	2012	Multiple environmental services as an opportunity for watershed restoration	Tow_2012_War	NA	4,080.00	-34.00	116.00	0	1	1	0	1	0	1	0	0	1	0	0	0	0	0	0	0	0	0	0	0	0	0	0	1	0	0	0	0	1	0	0	0	0	1	0	0	0
135	Trabucco et al.	Agriculture, Ecosystems and Environment	2008	Climate change mitigation through afforestation/reforestation: A global analysis of hydrologic impacts with four case studies	Tra 2008 Cha	NA	NA	-17.33	-66.14	0	0	1	0	0	0	1	0	0	1	0	0	0	0	0	0	0	0	0	0	0	0	0	0	0	0	0	1	0	1	0	0	0	0	0	1	0	0
					Tra_2008_Tun	NA	NA	-16.71	-65.66	1	0	0	0	1	0	1	0	0	1	0	0	0	0	0	0	0	0	0	0	0	0	0	0	1	0	0	0	0	1	0	0	0	0	0	1	0	0
					Tra_2008_Gua	NA	NA	-1.94	-78.71	0	0	1	0	0	0	1	0	0	1	0	0	0	0	0	0	0	0	0	0	0	0	0	0	0	1	0	0	0	1	0	0	0	0	0	1	0	0
					Tra_2008_Coa	NA	NA	-0.21	-80.11	0	0	1	0	0	0	1	0	0	1	0	0	0	0	0	0	0	0	0	0	0	0	0	0	0	0	0	1	0	1	0	0	0	0	0	1	0	0
					Tra_2008_GLOBE	NA	NA	NA	NA	1	0	0	1	1	0	1	0	0	1	0	0	0	0	0	0	0	0	0	0	0	0	0	0	1	1	1	0	0	1	0	0	0	0	0	0	0	0

Paper ID	Publication Reference				Study Information			Coordinates		Type of Intervention						Direct Metric		Direct Annual Water Yield Response				Baseflow			Indirect Water Yield Metrics									Forest Type				Data Acquisition Method				Continent					
	Author(s)	Publication name	Publication year	Publication title	Study case name	Years of discharge data	Catchment area (km^2)	Latitude	Longitude	Forest Restoration (explicit)	Forest Restoration (implicit)	Forestry	Afforestation/Forestation	Reforestation	Forest Regrowth	Water yield	Stream flow	Positive	Negative	No change	Unclear	Positive	Negative	Neutral	Increased Flooding/ Peak Flow	Decreased Flooding/ Peak Flow	Neutral Flooding/ Peak Flow	Increased GW level/ recharge	Decreased GW level/ recharge	Neutral GW level/ recharge	Increased soil infiltration / capacity	Decreased soil infiltration / capacity	Neutral soil infiltration / capacity	Native Forest	Non-native Forest	Mixed	ND Species	Field-Data	Model	Mixed	Historical Data/ Meta-data	Africa	Asia+ Middle East	Oceania	South+ Central America	Europe	North America
136†	Trimble & Weirich	Journal of Soil and Water Conservation	1987	Reforestation reduces streamflow in the southeastern United States	Tri_1987_Ocm	75	5,800.00	32.85	-83.62	0	0	0	0	1	0	1	1	0	1	0	0	0	0	0	0	0	0	0	0	0	0	0	0	0	0	0	1	1	1	1	1	0	0	0	0	0	1
					Tri_1987_Oco_M	71	7,640.00	33.08	-83.22	0	0	0	0	1	0	1	1	0	1	0	0	0	0	0	0	0	0	0	0	0	0	0	0	0	0	0	1	1	1	1	1	0	0	0	0	0	1
					Tri_1987_Fli	63	4,790.00	32.86	-84.10	0	0	0	0	1	0	1	1	0	1	0	0	0	0	0	0	0	0	0	0	0	0	0	0	0	0	0	1	1	1	1	1	0	0	0	0	0	1
					Tri_1987_Oco_G	71	2,820.00	33.57	-83.18	0	0	0	0	1	0	1	1	0	1	0	0	0	0	0	0	0	0	0	0	0	0	0	0	0	0	0	1	1	1	1	1	0	0	0	0	0	1
					Tri_1987_Cha_W	75	9,195.00	32.88	-85.17	0	0	0	0	1	0	1	1	0	1	0	0	0	0	0	0	0	0	0	0	0	0	0	0	0	0	0	1	1	1	1	1	0	0	0	0	0	1
					Tri_1987_Sav	75	19,450.00	32.09	-81.10	0	0	0	0	1	0	1	1	0	1	0	0	0	0	0	0	0	0	0	0	0	0	0	0	0	0	0	1	1	1	1	1	0	0	0	0	0	1
					Tri_1987_Tal	52	4,300.00	33.11	-85.53	0	0	0	0	1	0	1	1	0	1	0	0	0	0	0	0	0	0	0	0	0	0	0	0	0	0	0	1	1	1	1	1	0	0	0	0	0	1
					Tri_1987_Cha_N	73	3,030.00	33.98	-84.26	0	0	0	0	1	0	1	1	0	1	0	0	0	0	0	0	0	0	0	0	0	0	0	0	0	0	0	1	1	1	1	1	0	0	0	0	0	1
					Tri_1987_Sal_C	46	6,500.00	34.00	-81.06	0	0	0	0	1	0	1	1	0	1	0	0	0	0	0	0	0	0	0	0	0	0	0	0	0	0	0	1	1	1	1	1	0	0	0	0	0	1
					Tri_1987_Sal_S	39	4,200.00	34.18	-81.72	0	0	0	0	1	0	1	1	0	1	0	0	0	0	0	0	0	0	0	0	0	0	0	0	0	0	0	1	1	1	1	1	0	0	0	0	0	1
137	van Dijk et al.	Forest Ecology and Management	2007	Reforestation, water availability and stream salinity: A multi-scale analysis in the Murray-Darling Basin, Australia	Dij_2007_MBD	66	1,100,000.00	-37.10	147.77	0	1	1	0	1	0	1	0	0	1	0	0	0	0	0	0	0	0	0	0	0	0	0	0	0	0	0	0	0	1	0	1	0	0	1	0	0	0
138	van Lill et al.	Journal of Hydrology	1980	The effect of afforestation with Eucalyptus grandis hill ex maiden and Pinus patula schlect et cham. on streamflow from experimental catchments at Mokobulaan, Transvaal	Lil_1980_Mok_A	21	0.26	-25.28	30.57	0	0	1	1	0	0	1	1	0	1	0	0	0	0	0	0	0	0	0	0	0	0	0	0	0	1	0	0	1	0	0	0	1	0	0	0	0	0
					Lil_1980_Mok_B	21	0.35	-25.28	30.57	0	0	1	1	0	0	1	1	0	1	0	0	0	0	0	0	0	0	0	0	0	0	0	0	0	1	0	0	1	0	0	0	1	0	0	0	0	0
139	Vertessy et al.	Tree Physiology	1996	Long-term growth and water balance predictions for a mountain ash (Eucalyptus regnans) forest catchment subject to clear-felling and regeneration	Ver_1996_Pic	23	0.53	-37.90	145.36	0	0	1	0	0	1	1	0	0	1	0	0	0	0	0	0	0	0	0	0	0	0	0	0	1	0	0	0	1	1	1	0	0	0	1	0	0	0
140	Viola et al.	Water Resrouces Management	2014	Impacts of land-use changes on the hydrology of the Grande River Basin headwaters, Southeastern Brazil	Vio_2014_SRW	5	7,325.00	-22.00	-45.67	0	1	0	0	1	0	1	1	0	1	0	0	0	1	0	0	1	0	0	0	0	0	0	0	0	1	0	0	0	1	0	0	0	0	0	1	0	0
					Vio_2014_VRW	5	4,178.00	-22.33	-45.33	0	1	0	0	1	0	1	1	0	1	0	0	0	1	0	0	1	0	0	0	0	0	0	0	0	1	0	0	0	1	0	0	0	0	0	1	0	0
					Vio_2014_GRW	5	2,080.00	-21.67	-44.33	0	1	0	0	1	0	1	1	0	1	0	0	0	1	0	0	1	0	0	0	0	0	0	0	0	1	0	0	0	1	0	0	0	0	0	1	0	0
					Vio_2014_ARW	5	2,095.00	-22.20	-44.50	0	1	0	0	1	0	1	1	0	1	0	0	0	1	0	0	1	0	0	0	0	0	0	0	0	1	0	0	0	1	0	0	0	0	0	1	0	0
141	Vodogretskii	Soviet Meteorology and Hydrology	1976	Influence of agricultural afforestation on streamflow	Vod_1976_Vol_G	65	479.00	56.28	44.15	0	0	0	1	0	0	1	1	0	1	0	0	0	0	0	0	0	0	0	0	0	0	0	0	0	1	0	0	0	1	0	0	0	1	0	0	0	0
					Vod_1976_Vol_K	65	1,220.00	48.83	44.67	0	0	0	1	0	0	1	1	0	1	0	0	0	0	0	0	0	0	0	0	0	0	0	0	0	1	0	0	0	1	0	0	0	1	0	0	0	0
					Vod_1976_Vol_V	65	1,360.00	48.70	44.54	0	0	0	1	0	0	1	1	0	1	0	0	0	0	0	0	0	0	0	0	0	0	0	0	0	1	0	0	0	1	0	0	0	1	0	0	0	0
					Vod_1976_Oka	65	244.00	56.15	43.08	0	0	0	1	0	0	1	1	0	1	0	0	0	0	0	0	0	0	0	0	0	0	0	0	0	1	0	0	0	1	0	0	0	1	0	0	0	0
					Vod_1976_Kam	65	504.00	55.30	49.91	0	0	0	1	0	0	1	1	0	1	0	0	0	0	0	0	0	0	0	0	0	0	0	0	0	1	0	0	0	1	0	0	0	1	0	0	0	0
					Vod_1976_Ura_U	65	180.00	51.19	51.38	0	0	0	1	0	0	1	1	0	1	0	0	0	0	0	0	0	0	0	0	0	0	0	0	0	1	0	0	0	1	0	0	0	1	0	0	0	0
					Vod_1976_Ura_m	65	237.00	47.24	51.94	0	0	0	1	0	0	1	1	0	1	0	0	0	0	0	0	0	0	0	0	0	0	0	0	0	1	0	0	0	1	0	0	0	1	0	0	0	0
					Vod_1976_Don_G	65	695.00	50.97	39.50	0	0	0	1	0	0	1	1	0	1	0	0	0	0	0	0	0	0	0	0	0	0	0	0	0	1	0	0	0	1	0	0	0	1	0	0	0	0
					Vod_1976_Don_T	65	255.00	47.61	42.09	0	0	0	1	0	0	1	1	0	1	0	0	0	0	0	0	0	0	0	0	0	0	0	0	0	1	0	0	0	1	0	0	0	1	0	0	0	0
					Vod_1976_Don_m	65	422.00	47.24	39.79	0	0	0	1	0	0	1	1	0	1	0	0	0	0	0	0	0	0	0	0	0	0	0	0	0	1	0	0	0	1	0	0	0	1	0	0	0	0
					Vod_1976_Dne1_K	65	328.00	50.46	30.58	0	0	0	1	0	0	1	1	0	1	0	0	0	0	0	0	0	0	0	0	0	0	0	0	0	1	0	0	0	1	0	0	0	1	0	0	0	0
					Vod_1976_Dne1_m	65	500.00	46.65	32.70	0	0	0	1	0	0	1	1	0	1	0	0	0	0	0	0	0	0	0	0	0	0	0	0	0	1	0	0	0	1	0	0	0	1	0	0	0	0
					Vod_1976_Dne2_M	65	43.00	48.45	27.78	0	0	0	1	0	0	1	1	0	1	0	0	0	0	0	0	0	0	0	0	0	0	0	0	0	1	0	0	0	1	0	0	0	1	0	0	0	0
					Vod_1976_Dne2_n	65	72.10	46.55	29.87	0	0	0	1	0	0	1	1	0	1	0	0	0	0	0	0	0	0	0	0	0	0	0	0	0	1	0	0	0	1	0	0	0	1	0	0	0	0
					Vod_1976_Sou_m	65	63.70	47.04	31.86	0	0	0	1	0	0	1	1	0	1	0	0	0	0	0	0	0	0	0	0	0	0	0	0	0	1	0	0	0	1	0	0	0	1	0	0	0	0
					Vod_1976_Ob	65	252.00	55.01	82.94	0	0	0	1	0	0	1	1	0	1	0	0	0	0	0	0	0	0	0	0	0	0	0	0	0	1	0	0	0	1	0	0	0	1	0	0	0	0
					Vod_1976_Irt	65	1,643.00	66.60	67.02	0	0	0	1	0	0	1	1	0	1	0	0	0	0	0	0	0	0	0	0	0	0	0	0	0	1	0	0	0	1	0	0	0	1	0	0	0	0
142	von Stackelberg et al.	Transactions of the ASABE	2007	Simulation of the hydrologic effects of afforestation in the Tacuarembo River Basin, Uruguay	Sta_2007_D2	4	1.08	-31.27	-55.70	0	0	1	1	0	0	1	0	0	1	0	0	0	0	0	0	0	0	0	0	0	0	0	0	0	1	0	0	0	1	0	0	0	0	0	1	0	0
143	Wang et al.	Plant Biosystems	2012	Simulated water balance of forest and farmland in the hill and gully region of the Loess Plateau in China	Wan_2012_Yan	1	46.90	36.50	109.57	0	0	0	1	0	0	1	0	0	1	0	0	0	0	0	0	0	0	0	0	0	0	0	0	0	1	0	0	1	1	1	0	0	1	0	0	0	0
144	Wang et al.	Journal of the Science of Food and Agriculture	2012	Effect of reforestation on nitrogen and phosphorus dynamics in the catchment ecosystems of subtropcial China: The example of the Hanjiang River basin	Wan_2012_REG	11	159,000.00	32.02	106.70	0	1	0	0	1	0	1	0	0	0	1	0	0	0	0	0	0	0	0	0	0	0	0	0	0	0	0	0	0	1	0	0	0	1	0	0	0	0
145	Wang et al.	Proceedings of the XXV IUGG General Assembly, Melbourne, June-July 2011) (IAHS Publ. 353)	2012	Comparison between a statistical approach and paired catchment study in estimating water yield response to afforestation	Wan_2012_REV	55	0.03 - 102.00	NA	NA	0	0	0	1	0	0	1	0	0	1	0	0	0	0	0	0	0	0	0	0	0	0	0	0	0	0	0	0	1	0	0	1	0	0	0	0	0	0
146	Wang et al.	Journal of the American Water Resources Association	2008	Water-yield reduction after afforestation and related processes in the semiarid Liupan Mountains, northwest China	Wan_2008_Die	NA	0.25	35.47	106.33	0	1	0	1	1	0	1	0	0	1	0	0	0	0	0	0	0	0	0	0	0	0	0	0	0	0	0	0	1	1	1	0	0	1	0	0	0	0
147	Wangpimool et al.	International Soil and Water Conservation Research	2013	The effect of reforestation on stream flow in Upper Nan river basin using Soil and Water Assessment Tool (SWAT) model	Wan_2013_Nan	9	5,663.00	18.81	100.79	0	1	0	0	1	0	0	0	0	0	0	0	0	0	0	0	0	0	0	0	0	0	0	0	0	0	0	0	0	0	0	0	0	1	0	0	0	0
148	Waterloo et al.	Water Resouces Research	1999	Evaporation from Pinus caribaea plantations on former grassland soils under maritime tropical conditions	Wat_1999_Tul	1	NA	-18.00	177.45	0	0	1	1	0	0	1	0	0	1	0	0	0	0	0	0	1	0	0	0	0	0	0	0	0	0	0	0	1	1	1	0	0	0	1	0	0	0
					Wat_1999_Kor	1	NA	-18.02	177.30	0	0	1	1	0	0	1	0	0	1	0	0	0	0	0	0	1	0	0	0	0	0	0	0	0	0	0	0	1	1	1	0	0	0	1	0	0	0
149	Wattenbach et al.	Journal of Hydrology	2007	Hydrological impact assessment of afforestation and change in tree-species composition - A regional case study for the Federal State of Brandenburg (Germany)	Wat_2007_REG	11	280.00 - 80,258.00	52.36	13.01	0	1	0	1	0	0	0	0	0	0	0	0	0	0	0	0	0	0	0	0	0	0	0	0	0	0	0	0	0	0	0	0	0	0	0	0	1	0
150	Webb & Jarrett	Forest Ecology and Management	2013	Hydrological response to wildfire, integrated logging and dry mixed species eucalypt forest regeneration: The Yambulla experiment	Web_2013_Gee	16	0.80	-37.33	149.58	0	1	0	0	0	1	1	1	1	0	0	0	0	0	0	0	0	0	0	0	0	0	0	0	1	0	0	0	1	0	0	0	0	0	1	0	0	0
151	Webb & Kathuria	Journal of Hydrology	2012	Reponse of streamflow to afforestation and thinning at Red Hill, Murray Darling Basin, Australia	Web_2012_Red	20	1.95	-35.01	148.34		0	1	1	0	0	1	1	0	1	0	0	0	0	0	0	0	0	0	0	0	0	0	0	0	1	0	0	1	1	1	0	0	0	1	0	0	0
152	Webb et al.	Journal of Hydrology	2012	Longer-term changes in streamflow following logging and mixed species eucalypt forest regeneration: The Karuah experiment	Web_2012_Cor	35	0.41	-32.19	151.72	0	0	1	0	0	0	1	1	0	1	1	0	0	0	0	0	0	0	0	0	0	0	0	0	1	0	0	0	1	0	0	0	0	0	1	0	0	0
					Web_2012_Jac	35	0.13	-32.19	151.72	0	0	1	0	0	0	1	1	1	1	0	0	0	0	0	0	0	0	0	0	0	0	0	0	1	0	0	0	1	0	0	0	0	0	1	0	0	0
					Web_2012_Kok	35	0.97	-32.19	151.72	0	0	1	0	1	0	1	1	0	1	0	0	0	0	0	0	0	0	0	0	0	0	0	0	0	1	0	0	1	0	0	0	0	0	1	0	0	0
					Web_2012_Coa	35	0.38	-32.19	151.72	0	0	1	0	1	0	1	1	0	1	0	0	0	0	0	0	0	0	0	0	0	0	0	0	0	1	0	0	1	0	0	0	0	0	1	0	0	0
153	Wegehenkel	Physics and Chemistry of the Earth	2003	Longterm evaluation of land use changes on catchment water balance - a case study from North-East Germany	Weg_2003_Sto	44	220.00	52.58	14.13	0	0	0	1	0	0	0	0	0	0	0	0	0	0	0	0	0	0	0	0	0	0	0	0	0	0	0	0	0	0	0	0	0	0	0	0	1	0
154	Wine & Zou	Forest Ecology and Management	2012	Long-term streamflow relations with riparian gallery forest expansion into tallgrass prairie in the Southern Great Plains, USA	Win_2012_Cou	54	78.00	36.17	-96.88	0	0	0	0	1	0	0	0	0	0	0	0	1	0	0	0	0	0	0	0	0	0	0	0	0	0	0	0	0	0	0	0	0	0	0	0	0	1
155	Yu et al.	Geophysical Research Letters	2009	Quantitative estimate of water yield reduction caused by forestation in a water-limited area in northwest China	Yu_2009_Xia	NA	43.50	35.50	106.25	0	1	1	1	0	0	1	0	0	1	0	0	0	0	0	0	0	0	0	0	0	0	0	0	0	0	0	0	0	1	0	0	0	1	0	0	0	0
156	Yuan & Ren	Proceedings of a Symposium in Guangzhou, China. IAHS Publ. 319.	2006	Evaluating the influence of land-cover change on catchment hydrology through the modified Xinanjiang model	Yua_2006_Han	6	9,349.00	32.05	107.09	0	0	0	1	0	0	1	0	0	1	0	0	0	0	0	0	1	0	0	0	0	0	0	0	0	0	0	1	0	1	0	0	0	1	0	0	0	0
157†	Zhang et al.	Hydrology and Earth System Sciences	2012	Predicting effects of plantation expansion on streamflow regime for catchments in Australia	Zha_2012_Adj	13	391.00	-35.02	148.25	0	0	1	0	1	0	1	1	0	1	0	0	0	0	0	0	0	0	0	0	0	0	0	0	0	1	0	0	1	1	1	0	0	0	1	0	0	0
					Zha_2012_Bat	8	16.64	-33.32	116.57	0	0	1	0	1	0	1	1	0	1	0	0	0	0	0	0	0	0	0	0	0	0	0	0	0	1	0	0	1	1	1	0	0	0	1	0	0	0
					Zha_2012_Bom	10	559.00	-37.00	149.38	0	0	1	0	1	0	1	1	0	1	0	0	0	0	0	0	0	0	0	0	0	0	0	0	0	1	0	0	1	1	1	0	0	0	1	0	0	0
					Zha_2012_Bur	4	0.60	-35.13	138.70	0	0	1	0	1	0	1	1	0	1	0	0	0	0	0	0	0	0	0	0	0	0	0	0	0	1	0	0	1	1	1	0	0	0	1	0	0	0
					Zha_2012_Cra	5	606.00	-37.98	141.46	0	0	1	0	1	0	1	1	0	1	0	0	0	0	0	0	0	0	0	0	0	0	0	0	0	1	0	0	1	1	1	0	0	0	1	0	0	0
					Zha_2012_Dar	5	760.00	-38.15	141.77	0	0	1	0	1	0	1	1	0	1	0	0	0	0	0	0	0	0	0	0	0	0	0	0	0	1	0	0	1	1	1	0	0	0	1	0	0	0
					Zha_2012_Del	10	1,135.70	-36.98	149.05	0	0	1	0	1	0	1	1	0	1	0	0	0	0	0	0	0	0	0	0	0	0	0	0	0	1	0	0	1	1	1	0	0	0	1	0	0	0
					Zha_2012_Eum	7	502.00	-38.26	141.94	0	0	1	0	1	0	1	1	0	1	0	0	0	0	0	0	0	0	0	0	0	0	0	0	0	1	0	0	1	1	1	0	0	0	1	0	0	0
					Zha_2012_Goo	18	673.00	-35.19	148.20	0	0	1	0	1	0	1	1	0	1	0	0	0	0	0	0	0	0	0	0	0	0	0	0	0	1	0	0	1	1	1	0	0	0	1	0	0	0
					Zha_2012_Jin	9	390.00	-35.53	147.41	0	0	1	0	1	0	1	1	0	1	0	0	0	0	0	0	0	0	0	0	0	0	0	0	0	1	0	0	1	1	1	0	0	0	1	0	0	0
					Zha_2012_Pin	11	3.20	-37.29	145.05	0	0	1	0	1	0	1	1	0	1	0	0	0	0	0	0	0	0	0	0	0	0	0	0	0	1	0	0	1	1	1	0	0	0	1	0	0	0
					Zha_2012_Red	4	1.95	-35.12	149.35	0	0	1	0	1	0	1	1	0	1	0	0	0	0	0	0	0	0	0	0	0	0	0	0	0	1	0	0	1	1	1	0	0	0	1	0	0	0
					Zha_2012_Tra	6	89.00	-38.32	146.53	0	0	1	0	1	0	1	1	0	1	0	0	0	0	0	0	0	0	0	0	0	0	0	0	0	1	0	0	1	1	1	0	0	0	1	0	0	0
					Zha_2012_Den	4	243.00	-34.70	117.22	0	0	1	0	1	0	1	1	0	1	0	0	0	0	0	0	0	0	0	0	0	0	0	0	0	1	0	0	1	1	1	0	0	0	1	0	0	0
					Zha_2012_Yat	4	56.32	-33.70	117.29	0	0	1	0	1	0	1	1	0	1	0	0	0	0	0	0	0	0	0	0	0	0	0	0	0	1	0	0	1	1	1	0	0	0	1	0	0	0
158	Zhang et al.	Hydrological Processes	2008	Modelling the impact of afforestation on average annual streamflow in the Loess Plateau, China	Zha_2008_MANY	NA	110,000.00	38.00	110.00	0	1	1	1	0	0	1	1	0	1	0	0	0	0	0	0	0	0	0	0	0	0	0	0	0	0	0	0	1	1	1	0	0	1	0	0	0	0
159	Zhang et al.	International Journal of Sediment Research	2010	Effects of landuse change on surface runoff and sediment yield at different watershed scales on the Loess Plateau	Zha_2010_Leg	18	12.01	34.55	105.72	0	0	0	0	1	0	1	0	0	1	0	0	0	0	0	0	0	0	0	0	0	0	0	0	0	0	0	1	1	0	0	0	0	1	0	0	0	0
					Zha_2010_Qzd	18	1.09 - 1.36	34.62	105.73	0	0	0	0	1	0	1	0	0	1	0	0	0	0	0	0	0	0	0	0	0	0	0	0	0	0	0	1	1	0	0	0	0	1	0	0	0	0
					Zha_2010_Lyg	18	72.79	34.60	105.01	0	0	0	0	1	0	1	0	0	1	0	0	0	0	0	0	0	0	0	0	0	0	0	0	0	0	0	1	1	0	0	0	0	1	0	0	0	0
160	Zhang et al.	Journal of the American Water Resources Association	2008	Potential impact of afforestation on water yield in the subalpine region of southwestern China	Zha_2008_Miy	6	2,541.00	31.58	102.81	0	1	0	1	0	0	1	1	0	1	0	0	0	0	0	0	0	0	0	0	0	0	0	0	0	0	0	0	1	1	1	0	0	1	0	0	0	0
151	Zhao et al.	Hydrological Processes	2012	Changes in streamflow regime following vegetation changes from paired catchments	Zha_2012_CPIII	28	1.42	-29.00	29.25	0	0	1	1	0	0	1	1	0	1	0	0	0	0	0	0	0	0	0	0	0	0	0	0	0	1	0	0	1	1	1	0	1	0	0	0	0	0
					Zha_2012_GH2	20	3.10	-45.71	169.75	0	0	1	1	0	0	1	1	0	1	0	0	0	0	0	0	0	0	0	0	0	0	0	0	0	1	0	0	1	1	1	0	0	0	1	0	0	0
					Zha_2012_Red	15	1.95	-35.32	149.14	0	0	1	1	0	0	1	1	0	1	0	0	0	0	0	0	0	0	0	0	0	0	0	0	0	1	0	0	1	1	1	0	0	0	1	0	0	0
162	Zhao et al.	Forest Systems	2015	Effect of climatic change and afforestation on water yield in the Rocky Mountain Area of North China	Zha_2015_Cha	33	8,006.23	41.25	115.75	0	1	0	1	0	0	1	0	0	1	0	0	0	0	0	0	0	0	0	0	0	0	0	0	0	1	0	0	1	1	1	0	0	1	0	0	0	0
					Zha_2015_Bai	33	7,083.73	40.58	115.50	0	1	0	1	0	0	1	0	0	1	0	0	0	0	0	0	0	0	0	0	0	0	0	0	0	1	0	0	1	1	1	0	0	1	0	0	0	0
163	Zheng et al.	Journal of Hydrology	2008	Impacts of reforestation approaches on runoff control in the hilly red soil region of Southern China	Zhe_2008_Yan	3	NA	27.08	112.30	0	1	0	0	1	0	1	0	0	1	0	0	0	0	0	0	0	0	0	0	0	0	0	0	0	0	0	0	1	0	0	0	0	1	0	0	0	0
164	Zhou et al.	Forest Ecology and Management	2002	Hydrological impact of reafforestation with eucalipts and indigenous species: a case study in Southern China	Zho_2002_Xia_2	9	0.04	21.13	110.90	0	1	1	0	1	0	1	0	0	1	0	0	0	0	0	0	0	0	0	0	0	0	0	0	0	1	0	0	1	0	0	0	0	1	0	0	0	0
					Zho_2002_Xia_3	9	0.06	21.13	110.90	0	1	1	0	1	0	1	0	0	1	0	0	0	0	0	0	0	0	0	0	0	0	0	0	1	1	1	0	1	0	0	0	0	1	0	0	0	0
165	Zhou et al.	Water Resources Research	2010	Forest recovery and river discharge at the regional scale of Guangdong Province, China. Large scale assessment	Zho_2010_REG	50	179,752.00	23.50	113.00	0	1	0	0	1	0	1	0	0	0	1	0	1	0	1	0	0	0	0	0	0	0	0	0	0	0	0	0	1	1	1	0	0	1	0	0	0	0
166	Rodrigues et al.	Sociedade & Natureza	2015	Application of SWAT Model in the evaluation of water consumption in planted forest areas in Para river basin, Upper Sao Francisco, in Minas Gerais	Rod_2015_Par	32	12,300.00	-19.21	-45.14	0	0	1	0	1	0	1	0	0	1	0	0	0	0	0	0	0	0	0	0	0	0	0	0	0	1	0	0	0	1	0	0	0	0	0	1	0	0
167	Pereira et al.	Revista Brasileira de Ciência do Solo	2014	Impacts of Deforestation on Water Balance Components of a Watershed on the Brazilian East Coast	Per_2014_Gal	6	943.00	-20.32	-40.65	0	1	0	0	1	1	1	0	0	1	0	0	1	0	1	0	1	0	0	0	0	0	0	0	1	0	0	0	0	1	0	0	0	0	0	1	0	0

† Publication used for meta-analyses
