# Supplementary material for: Impacts of forest restoration on water yield: A systematic review
Source: PLoS One. 2017 Aug 17;12(8):e0183210. doi: 10.1371/journal.pone.0183210 (PMC5560669; doi:10.1371/journal.pone.0183210)
Supplement: S2 Table — Summary of data extracted from a sub-set of publications for the meta-analyses. The paper ID column corresponds to the same column in S1 Table. (PDF) [file pone.0183210.s002.pdf]

Table S2. Database for meta-analyses

Paper ID	Study case name	Catchment area (km^2)	Forest cover (%)	Peridod of discharge data		Water yield response (%)
				Start year	End year	
11	Bir_2014_Coa	1.50	90.00	1967	2011	NA
12	Bor_1988_Pad	1.96	81.00	1978	1986	-40
20	But_1994_Gan	262.00	8.90	1945	1993	-50
53	Hor_1975_Hub	0.16	33.00	1966	1972	-41
54	Hua_2004_Jia	1117.00	NA	1967	1989	-32
78	Li_2014_Lia	579.00	23.00	1971	2009	-6
79	Lim_1978_catch_9	0.12	80.00	1958	1971	NA
79	Lim_1978_catch_8	0.19	80.00	1958	1971	NA
82	Liu_2015_Mei	6983.20	71.00	1957	2006	-55
100	Mwe_1994_Luc	13.30	93.00	1961	1978	NA
107	Pit_1978_Que	200.00	55.00	1948	1971	NA
107	Pit_1978_Kla	NA	NA	1938	1971	NA
136	Tri_1987_Ocm	5800.00	27.50	1900	1975	-9
136	Tri_1987_Oco_M	7640.00	27.50	1904	1975	-15
136	Tri_1987_Fli	4790.00	25.70	1912	1975	-12
136	Tri_1987_Oco_G	2820.00	21.30	1904	1975	-21
136	Tri_1987_Cha_W	9195.00	20.00	1900	1975	-7
136	Tri_1987_Sav	19450.00	15.40	1900	1975	-15
136	Tri_1987_Tal	4300.00	11.80	1923	1975	-5
136	Tri_1987_Cha_N	3030.00	11.00	1902	1975	-6
136	Tri_1987_Sal_C	6500.00	10.50	1929	1975	-16
136	Tri_1987_Sal_S	4200.00	9.70	1927	1966	-16
157	Zha_2012_Adj	391.00	30.80	1995	2008	-32
157	Zha_2012_Bat	16.64	19.00	2000	2008	-60
157	Zha_2012_Bom	559.00	26.80	1990	2000	-66
157	Zha_2012_Bur	0.60	67.00	2003	2007	-100
157	Zha_2012_Cra	606.00	24.18	2004	2009	-53
157	Zha_2012_Dar	760.00	13.30	2004	2009	-44
157	Zha_2012_Del	1135.70	14.00	1990	2000	-27
157	Zha_2012_Eum	502.00	19.84	2001	2008	-25
157	Zha_2012_Goo	673.00	8.32	1990	2008	-49
157	Zha_2012_Jin	390.00	27.50	1996	2005	-32
157	Zha_2012_Pin	3.20	88.00	1998	2009	-100
157	Zha_2012_Red	1.95	78.00	2001	2005	-100
157	Zha_2012_Tra	89.00	58.00	1993	1999	-23
157	Zha_2012_Den	243.00	15.17	2004	2008	-90
157	Zha_2012_Yat	56.32	33.57	2004	2008	-58
